# Fuzzy Vaults in Biometric Cryptosystems: A Survey of Techniques, Performance, and Applications

**DOI:** 10.3390/s26092825

**Published:** 2026-05-01

**Authors:** Faria Farheen, Woo Yeol Yang, Sparsh Sharma, Saurabh Singh

**Affiliations:** 1 Department of Computer Science & Engineering, National Institute of Technology Srinagar, Hazratbal, Jammu & Kashmir, Srinagar 190006, India; faria_2023mcsc010@nitsri.ac.in (F.F.); sparsh.sharma@nitsri.ac.in (S.S.); 2 Cyber Warfare Technology Research Center, Cyber Security Research Division, Electronics and Telecommunications Research Institute (ETRI), Daejeon 34129, Republic of Korea; yotop93@naver.com; 3 AI and Big Data, Endicott College, Woosong University, Daejeon 34606, Republic of Korea

**Keywords:** biometric, cryptosystem, fuzzy vault, iris, sensing technology

## Abstract

Biometric sensing systems enable accurate identity recognition using unique physiological traits. These systems can be unimodal (single trait) or multimodal (multiple traits, such as iris and fingerprint). Biometric templates, digital representations of these traits, enhance security over traditional methods but are vulnerable to attacks. Unlike passwords, compromised templates cannot be replaced, necessitating robust protection. Various security schemes exist, including cancellable biometrics, biometric cryptosystems, sensing technology, and biometrics in the encrypted domain. Cancellable biometrics apply transformations, such as biometric salting, to obscure the original data. Biometric cryptosystems integrate cryptographic techniques, including key generation and key binding, to enhance security. Biometrics in the encrypted domain, such as homomorphic encryption, ensures data remains encrypted during storage and computation. This survey focuses on the fuzzy vault method, a key-binding biometric cryptosystem. It analyses its applications, security performance, and associated challenges across different domains. By analysing advancements in fuzzy vault mechanisms, this paper provides insights into enhancing sensor-based biometric security. The study aims to serve as a reference for researchers exploring secure and efficient biometric authentication methods, ensuring robust protection against unauthorised access while maintaining the integrity and usability of biometric data in real-world applications.

## 1. Introduction

Authentication has been the foundational feature of data and service security for the past 6 decades. The first authentication was presented by Fernando Corbató, who resolved a security issue by using passwords to authenticate users. The authentication mechanism is a process in which the user enters a value “x” into the system, the system computes “f(x) = y,” and compares “y” to the previously stored value to verify the user’s identity. This authentication mechanism has evolved from PIN- and password-based systems to multi-factor authentication, which is still evolving to provide stronger security for the data currently stored in online formats.

With the advancement in technology, the traditional use of PINs and passwords became less reliable at providing security against attacks that have become increasingly easy to execute. The mishandling and numerous leaks of password databases led to the next step in improving the authentication process: the inclusion of multi-factor authentication, combining multiple authentication mechanisms to provide a highly secure environment for protecting online data and services.

Multi-factor authentication combines knowledge-based, possession-based, and biometric (inherence-based) factors: knowledge-based authentication, where the user has to remember a password; the possession-based authentication mechanism, where the user must have tokens or cards to prove their identity to the system; and lastly, inherence-based authentication, which includes the biometric traits of the individual, like fingerprint and retinal scans, which are unique to the individual. Even though multi-factor authentication is highly secure in protecting against identity theft, it remains challenging due to its complex implementation and higher hardware costs. Nevertheless, biometric authentication, especially fingerprint scanning, is increasingly being used with multi-factor authentication, which has been proven to improve identity proofing.

**Figure 1:**
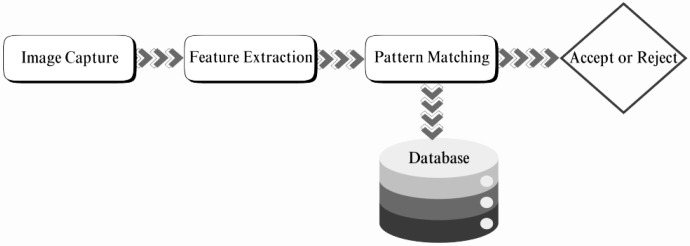
Biometric cryptosystem: illustration of verification.

Biometric traits are unique to individuals. They encompass a wide range of features, including voice biometrics, facial recognition, and eye recognition, as well as hand geometry, fingerprint scanners, and thermal image recognition. Voice recognition analyses pitch and tone for user verification; however, it is generally less secure than other biometric modalities. The facial recognition system analyses the spacing between facial features, shape, and shadows to authenticate individuals; however, it is vulnerable to spoofing attacks using facial images. Fingerprint authentication is vulnerable to fingerprint feature and pattern theft, as fingerprints can be collected from surfaces the individual comes into contact with. Eye recognition, or the iris recognition system, identifies the patterns in the iris region of the pupil, which are very difficult to replicate. As shown in Figure 1, a typical biometric cryptosystem involves capturing the image, extracting features, matching the query image against the stored pattern, and, based on the match, accepting or rejecting a user.

The factors used for the authentication are evaluated based on some characteristics, which are given as follows:

○ Universality specifies that the factor selected for authentication must be universally present in every individual.
○ Uniqueness indicates that the factor must be exclusively present with the individual so that individuals may be easily recognisable and distinguishable from one another.
○ The selected feature must be easy to acquire in practical conditions, maintaining the efficacy and practicality of the collection method.

The biometric characteristics selected for the authentication must be precise, efficient, and robust. The other characteristic to consider is how well people will accept technology in day-to-day life. The most important characteristic to consider when selecting a trait is the difficulty of collecting and spoofing a sample of the feature [1].

### 1.1. Iris as a Biometric Feature

Among the biometric characteristics discussed for secure authentication, the iris is among the most widely used and reliable traits. The iris is the coloured region of the eye that regulates pupil size. It is responsible for regulating the amount of light entering the retina. Since the iris is an internal structure, it is less affected by environmental factors, making it a reliable biometric trait [2].

The iris is highly distinctive, as no two individuals share identical iris patterns. This distinctiveness satisfies the uniqueness requirement of biometric systems [3]. Even identical twins have distinct iris patterns due to epigenetic variation and developmental differences. The intricacy and sophistication of iris patterns provide a rich set of features for biometric matching, thereby enhancing authentication reliability. This authentication trait also provides lifelong stability, as it does not wear down or fade over time, unlike other features such as fingerprint or facial recognition. This characteristic also provides the longstanding efficiency of the recognition system [4].

These properties of stability, reliability, and uniqueness make the iris a valuable feature widely used for identification across domains such as anti-terrorism and border control, as well as in government ID systems. It has applications across premises access control (such as in homes, offices, and laboratories), secure financial transactions (such as bank account access and credit card authentication), internet security, and healthcare and patient identification [5].

Working on the iris recognition system requires specific steps to ensure accurate functionality. The process is composed mainly of two steps [3]. First is the enrolment phase, where a high-quality camera is employed to capture a detailed image of the iris; then, the features of the iris are extracted utilising any of the numerous methods that are proposed, like Daughman’s method [6], the Gabor Filter method [7], wavelet transformation [8], and now, convolutional networks [9] are also being implemented for this purpose. The extracted modalities capture distinct textural patterns, including ridges, furrows, and crypts [3]. These features are then logically encoded, again utilising one of the multiple methods, such as the Gabor Wavelet Transform [4], Log-Gabor Transform [10], Discrete Cosine Transform (DCT) [11], etc., into a digital representation known as a biometric template, which is then stored in a database. The second phase comprises the verification phase, during which the query sample is analysed and matched for identification. First, the query data point is captured; then, the iris pattern is extracted using the same mechanism as in the enrolment phase; and finally, the extracted pattern is analysed and compared with the stored template. If the new pattern matches the template, the individual is authenticated and considered to be legitimate [3].

### 1.2. Need for Securing Biometric Features

The biometric templates are vulnerable to various software attacks, such as inversion, replay, and substitution attacks, whether stored in the database or in transit for computational purposes. If the template is compromised, it results in the irreversible loss of crucial data. However, the protection of the biometric template is often neglected due to unawareness of attack methods, the misconception that biometric security mechanisms are more difficult to break than traditional passwords, and a lack of awareness among users and researchers about protecting the template. In addition, overlooking the challenges of performance and security in biometric systems further contributes to this oversight.

**Figure 2:**
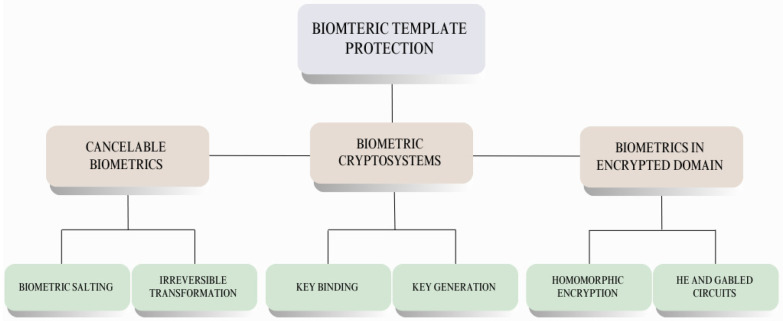
Taxonomy of biometric template protection schemes.

For this purpose, researchers have proposed numerous methods and algorithms, as depicted in Figure 2, to provide security, encrypt the biometric template, and protect it from unauthorised access and interpretation [12].

The three focal classifications of the biometric template protection procedures are depicted in Figure 2 and discussed in the following section.

1. **Cancellable biometrics (CB)**: A mechanism wherein the biometric template, like that of the fingerprint, is converted into a secure modified form by using mathematical transformations. To make the transformation unique to each individual, a secret key or user-specific data is also added, and the modified version, along with the user key, is stored for verification. The system then applies the same transformation to the query template and compares the transformed version with the stored version during authentication. If security is compromised, the transformed version is replaced, just as the password is, without affecting the users’ actual biometric template features.
  The cancellable biometric approach may follow a many-to-one transformation, combining multiple biometric features from the same individual, though somewhat different, into a single modified biometric template. This method prevents reverse engineering of the original biometric, as even if an intruder acquires the transformed biometric, they cannot recreate the individual’s original biometric feature.
  Another mechanism for cancellable biometrics is the salting approach, in which the user-specified key, such as a password, is combined with the biometric feature data to produce a secure template.
2. **Biometric in an encrypted domain**: This is yet another mechanism for securing biometric templates by performing all the computations and processing on the data in an encrypted form. This method ensures that the data remains secure even during computations and transmission. It predominantly utilises two cryptographic techniques:
  - **Homomorphic encryption (HE)**: The mathematical operations, like matching the query template with the features, take place on the encrypted data without ever decrypting the encrypted template, ensuring that the biometric template is protected even when computations are being performed on it.
  - **Garbled circuits**: This method permits a secure multi-party computation, which signifies that multiple parties can perform computations on the encrypted biometric data in conjunction without even revealing the actual biometrics.
3. **Biometric cryptosystems (BC)**: To enhance security, this approach integrates biometrics and cryptography. It follows a computational approach to encrypting biometric data. Instead of storing the raw data directly in the database, it encrypts the data and stores the secure sketch (error-tolerant representation of biometric data used for key recovery) along with the cryptographic key. Unlike the cancellable biometric, where the key used is user-specified, the key in the biometric cryptosystem is essentially either derived from the template itself or randomly generated. This encrypted biometric template and the key are known as auxiliary data. When the authentication process starts, the system, instead of matching the biometric template features, validates the authenticity of the key retrieved from the query template by comparing it with the cryptographic key stored in the database. Error-correcting codes are also used to account for natural variations in biometric data samples. Two prominent methods used in the biometric cryptosystems are as follows:
  - **Key-binding mechanism**: A secret key is randomly generated and securely affixed to the biometric data. During authentication, only the correct set of biometric features can retrieve the key. This randomness of the key prevents its disclosure. Even if biometric data is somehow stolen, the key remains unknown, thereby enhancing security. This mechanism also works well with biometric data that has some distortion due to positioning or lower exposure to light, due to the incorporation of error correction codes. Two frequently used key-binding methods are the fuzzy vault mechanism, which locks the key to a biometric template, and the fuzzy commitment mechanism, which uses mathematical functions to bind the key to biometric data.
  - **Key-generation mechanism**: Here, the key is generated directly from biometric data, without requiring it to be stored separately in the database. It is intolerant of biometric variations, and if biometric data is stolen, the key is compromised as well. The prominent method used in the key-generation mechanism is the fuzzy extractor, which produces repeatable and stable keys while handling small variations in the biometric data and secure sketches that are known to extract key-like features from the biometrics [12].

In biometric-based systems used to secure private keys, two notable methods are the biometric key release system and the biometric encryption system. The biometric key release mechanism often stores the private keys in the database. It releases them only after efficient authentication is achieved by matching the stored template with the query template. However, if the database is compromised, it exposes the templates and security keys to theft, even if the templates are encrypted using the cancellable biometric procedure. Unlike the biometric key release mechanism, biometric encryption provides stronger security because it stores only the encrypted key and biometric data. Even if this auxiliary data is stolen, it is challenging for the attacker to decrypt and retrieve the original templates. The keys can only be accessed once a legitimate template is provided to the system.

Numerous biometric encryption mechanisms are available, but biometric cryptography provides a highly secure method for combining biometric templates with cryptographic keys [13].

In this survey, the main focus will be on biometric cryptosystems, particularly the fuzzy vault procedure, which falls under the key-binding mechanism.

Although conventional template protection schemes have demonstrated effectiveness in securing stored biometric data, contemporary developments in decentralised identity management and AI-driven attacks necessitate broadening the security perspective of these authentication systems. Securing data at the processing stage, supporting privacy-preserving search, and maintaining robustness against adversarial manipulation have therefore become essential requirements in modern distributed and cloud-based biometric systems. To address these needs, this work provides a structured comparison of classical and contemporary fuzzy vault approaches, covering feature extraction strategies, cryptographic enhancements, and performance metrics. Beyond reviewing existing techniques, this survey identifies emerging research challenges and potential future directions. It further extends prior surveys by incorporating decentralised identity frameworks, privacy-preserving encrypted search mechanisms, and considerations of adversarial robustness, thereby broadening the scope to next-generation biometric cryptosystems.

## 2. Fuzzy Vaults in Biometric Cryptosystems

Fuzzy vault is a cryptographic construction presented by Ari Juels and Madhu Sudan that leverages fuzzy data to secure actual data from intruders [14]. This biometric cryptosystem is chosen for this survey because it has been successfully applied in biometric protection schemes. This is one of the prominent biometric cryptographic techniques and a reliable method for securing biometric templates [13].

The mechanism uses a set of elements, “A”, to lock a secret that can only be unlocked by another set, “B”, which closely superimposes on “A”. Unlocking the vault through sufficient overlap between the sets guarantees error tolerance, meaning that slight variations in query biometric features do not prevent successful authentication. The order-invariance property (system performance is independent of feature ordering) further means that the order of feature elements does not affect system operation.

The encryption of the biometric template starts by selecting a random secret key “k” and applying a permutation function, preferably a polynomial, to it. Then, chaff points (random decoy points added to obscure genuine data) are added to the real points representing the actual biometric features. This is done to obscure the actual feature points from attackers. The database now contains the polynomial coordinates for both the actual and chaff points. The decryption process begins by acquiring the query template to be matched against the actual feature set. Then, the polynomial is reconstructed using the matched points from the query set, and the secret key “k`” is retrieved. The retrieved key “k`” is matched with the previously generated key “k”. Authentication is complete only when the keys match; otherwise, it fails. The security of the method depends on the complexity of reconstructing the polynomial [13].

### 2.1. Implementation of Fuzzy Vaults

The implementation of the fuzzy vault mechanism follows some crucial steps for the successful encryption of the biometric template:

#### 2.1.1. Feature Extraction

**Figure 3:**
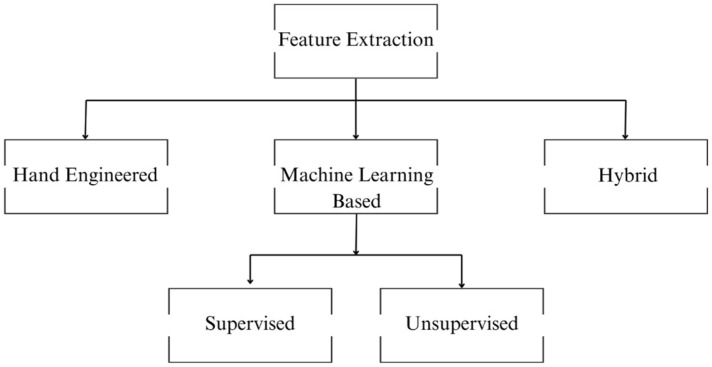
Feature extraction methods.

Three main methods, as shown in Figure 3, are explored for feature extraction: hand-engineered feature extraction, machine/deep learning-based feature learning, and hybrid approaches. Table 1 summarises the various approaches used to extract features across different modalities.

1. **Hand-Engineered Feature Extraction**: The extraction and representation of biometric features are done by crafting algorithms and mathematical formulations that are not digitalised. Thus, the design of these procedures necessitates domain expertise. This method is prominently utilised as it does not require a large dataset [15]. The algorithms are designed based on the biometric features to be extracted. There are various biometrics that can be used to authenticate a user, but the focus here will be on fingerprint, face, and iris.
  - **Fingerprint**: The methods developed for the extraction of features for fingerprints essentially emphasise the need to obtain some patterns from the fingerprints inherently, the points in the fingerprints where the crested lines terminate or cleave, called the minutiae points [16]. The spatial relationships that define how the patterns are affiliated with one another with respect to their positioning and containment are also considered features extracted from the fingerprints to authenticate the user [17]. Another feature used to verify the user is the orientation field, which indicates the direction in which the lines or ridges are pointing. It helps analyse the flow in the fingerprint pattern [18]. Various hand-engineered methods have been proposed to extract the aforementioned features.
    The feature extraction process includes the minutiae-based feature extraction method proposed by Ratha et al., which extracts fingerprint features from ridge terminations and bifurcations [19]. Jin et al. [20] proposed yet another method that makes use of wavelet and Fourier–Mellin transforms and acquires the invariant features. This method also makes the system resilient to rotations and scaling. The triangle formation method [21] constructs a triangle by using three minutiae points. Farooq et al. proposed that the comparative placement of the formed triangles generates a robust system that is flexible to distortion. The more recent proposed methods include the Minutia Cylinder Code (MCC), which was improved by Bedari et al. [22] and further enhanced the efficacy of cancellable fingerprint features; further, Sun et al. [23] proposed a pioneering technique where the fingerprint features are represented in a binary representation of consistent length, ensuring a standardised biometric template representation. The Scale-Invariant Feature Transform (SIFT) [24] is another method proposed by Djebli et al., in which minutiae points are extracted based on their positions, making the system transformation-invariant.
  - **Face**: Face biometrics have evolved recently and gained a lot of popularity due to their ease of use and efficiency, but securing the data associated with face recognition systems is crucial, as it is an irreversible template, and if compromised, it cannot be reconfigured. For this purpose, Savvides et al. [25] analysed facial images using the Fourier transformation that creates a matrix to capture the prime features of the face. Oh et al. [26] proposed a method where the facial features were extracted by compressing the horizontal and vertical anatomy of the face. A notable mapping mechanism, Baker’s mapping, as proposed by Faragallah et al. [27], generates distinctive filters for extracting facial features.
    Xu et al. [28] leveraged a dimensional PCA method based on quaternions, which improved the facial recognition method. With the advent of quantum computing, Alhumyani et al. analysed how it could be incorporated into biometric systems to represent facial features better [29]. A method where the image of the face is segregated into sections that are known as Voronoi patches was proposed by S. P. and Thomas [30], and the Log-Gabor filter is applied to extract the face’s salient features.
  - **Iris**: It has the highest accuracy rate among all biometrics in theory and practice and is considered to have higher recognition rates as well. The iris recognition system is successfully executed by obtaining high-definition iris images and then recognising the unique patterns within them. The extraction techniques used for this purpose predominantly prioritise examining the normalised iris images to certify the accuracy of the recognition system [15]. Before extracting the feature, a wavelet transform is applied to the image to generate bit codes from its textures and patterns [31].
    Zuo et al. proposed a method using a one-dimensional Log-Gabor filter and an autonomous segmentation algorithm to cipher the iris features [32]. Rathgeb et al. Captured the important features of the normalised iris images by applying the Log-Gabor filter row by row [33]. In 2017, Umer [34] used a statistical method, SIFT (Scale-Invariant Feature Transform) descriptors, to generate a global representation of the unique iris patterns obtained from the locally extracted iris patterns using normalised iris images.
2. **Machine Learning-based Feature Extraction**: This approach is explored as it provides a reliable mechanism to extract features and boosts the recognition systems’ performance. Two main approaches are exploited under this category of feature extraction: supervised machine learning, which works on the labelled data, and unsupervised learning, which works with the unlabelled datasets [15].
  - **Supervised Machine Learning Approaches**: Supervised machine learning models basically consist of layers of networks, each layer consisting of units or neurons. These complex networks can identify complex patterns and learn features from labelled datasets. The convolutional neural network (CNN) [35] and recurrent neural network (RNN) [36] are the most prominently used models. CNN models treat images as grid-like data and apply layers of convolutional and pooling operations, along with filters, to extract relevant and prominent features [37] (Goodfellow et al., *Deep Learning* textbook). Jang and Cho leveraged a general CNN and encoded the relevant features of the images into binary codes. They proposed a deep hash-based structure, called DTH (Deep Table-based Hashing), that enhances non-invertibility of face data through noise embedding (biometric salting) combined with intra-normalisation (normalisation applied within feature groups to reduce burst effects), which suppresses burst effects (over-representation of repetitive features) in the descriptor while maintaining classification performance [35]. To ensure data encryption, Abdellatef et al. combined multimodal biometrics with convolutional neural networks [38]. He incorporated a multi-biometric recognition system that used face and iris datasets, along with multiple CNN models, to extract features. Using multiple CNN models enabled feature extraction across facial regions. Sandhya et al. [39] proposed a CNN-based model incorporating triple loss and a cross-folding mechanism to implement a multi-instance iris recognition system. Sakr et al. [40] proposed the feature extraction of ECG datasets using the VGG16 CNN model, while Kim et al. [41] used the pre-trained model of ResNet 50 with a SoftMax Out fusion network to extract the features from face and periocular image datasets. Kim and Pyun [36] introduced real-time biometric recognition based on ECG datasets using recurrent neural networks.
  - **Unsupervised Learning Approaches**: The unsupervised learning mechanism makes use of unlabelled biometric datasets to learn unique patterns and features from them and then classify or recognise the query images based on the learned features. Tarek et al. [42] proposed a generative adversarial network (GAN) that leverages keyless biometric authentication for multi-instance biometric features. Sardar et al. [43] proposed a biometric authentication system using palmprint datasets, applying the K-means clustering technique to classify legitimate users correctly. E. Pintelas [44] and Siddhad et al. [45] both implemented convolutional autoencoders (CAE) to produce cancellable biometric authentication systems.
3. **Hybrid Approaches**: Hybrid feature extraction methods are a strong tool for fusing the advantages of both conventional hand-engineered methods and deep learning-based representations. In these techniques, handcrafted features such as SIFT, wavelet transforms, and texture descriptors are either extracted as a preprocessing step or used simultaneously with convolutional neural networks (CNNs), enabling the detection of both local structural information and high-level semantic patterns. For example, Scale-Invariant Feature Transform (SIFT), along with a CNN, was used successfully for Indian Sign Language recognition and showed a remarkable accuracy improvement, achieving 92.78% accuracy [46]. Palm vein [47] recognition systems have also adopted hand-crafted descriptors, such as the Histogram of Oriented Gradients (HOG) and the Discrete Wavelet Transform (DWT), combined with CNN-based architectures to improve recognition robustness and cross-dataset performance. Alternatively, vector quantisation [48] is utilised, and features extracted from Kekre’s Median Codebook and Kekre’s Fast Codebook (hand-engineered methods) are fused with features extracted using CNNs, aiming to achieve the best possible accuracy. Colour histogram-based descriptors are combined with CNN embeddings to enhance the discriminability of challenging biometric problems, such as person re-identification. A Feature Fusion Network (FFN) is created, in which features extracted using methods such as RGB, HSV, YCbCr, Lab, YIQ colour features, and Gabor texture features are mapped to CNN features to increase the accuracy of person re-identification systems [49]. These hybrid methods are highly valuable in limited-training-data scenarios because they offer the reliability of handcrafted descriptors while exploiting the flexibility of deep models—making them a great fit for problems like fuzzy vault construction that demand low-dimensional yet highly discriminative features.

**Table 1:** **Comparative analysis of feature extraction methods for biometric authentication and template protection.** Performance is evaluated using standard metrics, including False Acceptance Rate (FAR), False Rejection Rate (FRR), Genuine Acceptance Rate (GAR), and Equal Error Rate (EER).

Proposed By	Feature Extraction Method	Protection Scheme	Reported Performance	Dataset
Ratha et al. [19]	Minutiae-Based Feature Extraction, Hand-engineered	Cancellable + WSQ + challenge/response	False Acceptance Rate (FAR) ≈ 10^−6^, False Rejection Rate (FRR) ≈ 10^−4^ (verification); Security: 22–82 bits	Proprietary Modality Fingerprint
Jin et al. [20]	Wavelet and Fourier–Mellin Transforms, Hand-engineered	BioHashing (token-based)	Equal Error Rate (EER) = 0%	FVC 2002 (Set A) DB1, DB2, DB3 and DB4, Modality Fingerprint
Farooq et al. [21]	Robust Triangles Using Comparative Placement, Hand-engineered	Cancellable + permutation + MD5	EER = 0% (verification), 1.59% (lost token); FRR = 0% at FAR < 10^−3^	1000 fingerprints Modality Fingerprint
Bedari et al. [22]	Minutia Cylinder Code (MCC), Hand-engineered	Cancellable (index vectors + averaging)	EER = 0.04–3.28% range depends on dataset/key scenario	FVC2002 DB1-DB3, FVC2004 DB1–DB3 Modality Fingerprint
Sun et al. [23]	Binary Representation of Fingerprint Features, Hand-engineered	Cancellable (chaotic key + Bloom filter)	EER = 0.08–1.27% (different keys), 0.17–4.49% (same/lost-key scenario)	FVC2002 DB1–DB3, FVC2004 DB1–DB3 Modality Fingerprint
Djebli et al. [24]	Scale-Invariant Feature Transform (SIFT), Hand-engineered	Cancellable (random projection)	EER = 0% (different key), 1.78% (stolen key)	FVC 2002 DB1 Modality Fingerprint
Savvides et al. [25]	Fourier Transformation, Hand-engineered	Cancellable (random convolution kernel)	Verification rate = 100% (PSR metric; no EER/FAR/FRR reported)	CMU PIE Modality Face
Oh et al. [26]	Horizontal and Vertical Anatomy Compression, Hand-engineered	Cancellable (random projection + fusion)	EER ≈ 1–5% (user-specific token), ≈7–15% (shared/stolen token)	AR, FERET, ORL, Sheffield, and BERC Modality Face
Faragallah et al. [27]	Baker’s Mapping, Hand-engineered	Cancellable encryption	EER = 0%, FAR = 0%, FRR = 0.02%, Accuracy = 98.43%	AT&T, YALE, UFI, LFW, FERET Modality Face
Xu et al. [28]	Quaternion-Based Principal Component Analysis (PCA), Hand-engineered	Cancellable permutation	Accuracy = 98.85–99.00%	Aberdeen, GT, VIS, YMU Modality Face
Alhumyani et al. [29]	Quantum Representation of Facial Features, Hand-engineered	Cancellable (Hilbert permutation)	AROC = 0.9951 (average)	LFW, FERET, ORL Modality Face
S. P. and Thomas [30]	Voronoi Patches with Log-Gabor Filter, Hand-engineered	Cancellable (distortion)	EER = 1.38–1.76%; DI ≈ 1.35; RI ≈ 93.1%	CASIA-FaceV5 Modality Face
Zuo et al. [32]	1D Log-Gabor Filter with Autonomous Segmentation, Hand-engineered	Cancellable (non-invertible, revocable): row-shift + addition (GRAY-COMBO), row-shift + XOR (BIN-COMBO), random pattern ±× (GRAY-SALT), XOR w/random key (BIN-SALT)	ROC-based; no EER/FAR/FRR. GRAY-COMBO (260-bit) and GRAY-SALT: low FRR; BIN-COMBO (560-bit) degrades under noise.	MMU1 (100 images) Modality Iris
Rathgeb et al. [33]	Row-wise Log-Gabor Filtering, Hand-engineered	Cancellable hashing (Bloom filter, Ma et al. wavelet)	EER = 1.14–1.49%; FNMR at FMR = 0.01% reported	CASIA Iris V3 Modality Iris
Umer [34]	Global SIFT Descriptors, Hand-engineered	Modified BioHashing using two tokens	CRR = 100%; EER ≈ 0% (MMU1, UPOL, CASIA-v3, IITD, UBIRIS); CRR = 99.83%; EER = 0.43% (CASIA-syn)	MMU1, UPOL, CASIA-Interval-v3, IITD, UBIRIS.v1 and CASIA-syn. Modality Iris
Jang and Cho [35]	Deep Hash-Based Structure (DTH), Machine Learning-Based Supervised	Hashing-based cancellable	EER = 0.0048–0.0961%; mAP = 0.7416–0.9934	YouTubeFaces, FaceScrub Modality: Face
Kim and Pyun [36]	Real-time ECG Recognition using a Recurrent Neural Network (RNN) Machine Learning-Based Supervised	No template protection	Accuracy = 99.73–100%	MIT-BIH (NSR, ARR) Modality: ECG
Abdellatef et al. [38]	Multimodal CNN for Face and Iris Machine Learning-Based Supervised	Cancellable encryption	Accuracy = 97.38–98.93% (protected system)	FERET, LFW, PaSC Modality: Face+Iris
Sandhya et al. [39]	CNN with Triple Loss and Cross-Folding Machine Learning-Based Supervised	Cancellable (DNA encoding)	Authors report competitive performance relative to existing works; specific metrics not disclosed.	IITD and MMU Modality: Iris
Sakr et al. [40]	ECG Feature Extraction using VGG16 Machine Learning-Based Supervised	Cancellable hashing	EER = 0.40–0.44%; Accuracy = 98.60–98.85%	ECG-ID and PTB databases Modality: ECG
Kim et al. [41]	ResNet 50 with SoftMax Out Fusion Machine Learning-Based Supervised	Cancellable (GAN + XOR)	EER = 2.12% (Facescrubdataset), EER = 6.67% (average over six datasets)	AR, Ethnic, Facescrub, IMDBWiki, Pubfig, YTF Modality: Face
Tarek et al. [42]	GAN for Keyless Biometric Authentication Machine Learning-Based Unsupervised	Cancellable + cryptosystem	EER = 0.656%; Accuracy = 99.35%	CASIA Iris V3-Internal Modality: iris
Sardar et al. [43]	Palmprint Authentication with K-means Clustering Machine Learning-Based Unsupervised	Cancellable (PalmHashing) + biometric cryptosystem (Huffman coding + XOR encryption)	CPS3: 100% CRR (Bosphorus and CASIA, 20–100 bits) Face: 100% | Iris: 99.79% | Periocular: 98.83%	BOSPHORUS and CASIA-Palmprint Modality: palmprint
E. Pintelas et al. [44]	Convolutional Autoencoders Machine Learning-Based Unsupervised	None	Outperforms traditional approaches	Ultrasound images Modality: USG images
Dudhal [46]	SIFT, along with CNN Hybrid	None	Hybrid SIFT: 92.78% accuracy Adaptive thresholding: 91.84%	50-class Indian Sign Language dataset Modality: Hand Sign
Wulandari [47]	HOG and DWT with CNN Hybrid	None	Accuracy, AUC, EER CASIA: 99.85% | PUT: 98.12% | Vera: 95.57% Tongji: 94.91% | PolyU: 85.88%	CASIA, Vera, Tongji, PolyU, and PUT Modality: Palmprint
Sawant [48]	Vector Quantisation Hybrid, CNN + Kekre’s Median/Fast Codebook (KMCG/KFCG)	None	Accuracy, training time, parameter count Iris: 97.03% | Fingerprint: 95.99% Palmprint: 96.29% | Face: 96.86%	LFW, UPOL IITD, IITD V1, serSignatureDatabase Modalities: Iris, fingerprint, palmprint, face datasets, signature
S. Wu [49]	Colour Histogram-Based Descriptors, CNN Hybrid	None	Rank-1 accuracy (CMC curve) VIPeR: 51.06% | CUHK01: 55.51% | PRID450s: 66.62%	VIPeR, CUHK01, PRID450s Modalities: Human Body

Abbreviations: CRR: Correct Recognition Rate; FNMR: False Non-Match Rate; FMR: False Match Rate; mAP: mean Average Precision; PSR: Peak-to-Sidelobe Ratio; CMC: Cumulative Match Characteristic; DI: Decidability Index; RI: Recognition Index; AROC: Area under ROC Curve.

**Comparative Analysis of Feature Extraction Methods:**

A comparative analysis of the feature extraction techniques described in Table 1 identifies important considerations for the use of feature representations in the design of a fuzzy vault biometric cryptosystem. Fuzzy vault authentication protocols impose several requirements on feature representations that go beyond those typically required by standard authentication techniques. In particular, feature representations should support unordered sets, tolerate noise, and be robust to within-class variations. Hence, the applicability of feature extraction techniques should be determined not only by their ability to achieve high recognition rates but also by other factors.

Methods that incorporate fingerprint-based feature extraction consistently achieve strong verification performance across multiple studies. While minutiae-based feature extraction methods proposed by Ratha et al. [19] result in a False Acceptance Rate (FAR) of approximately 10^−6^ and a False Rejection Rate (FRR) of approximately 10^−4^, several studies report near-zero error rates, including Jin et al. [20] and Farooq et al. [21], although performance degrades when tokens are lost. In contrast, methods such as Bedari et al. [22] and Sun et al. [23] exhibit a wider EER range. Also, under stolen-key conditions, Djebli et al. [24] report EERs of 1.78% and 0% under different key conditions. It is observed that fingerprint features exhibit high discriminative capability only under controlled conditions; however, when integrated into fuzzy vault constructions, their sensitivity to alignment and key conditions must be carefully addressed.

On the other hand, due to illumination, pose, and changes in expression, face-based feature extraction is more variable. Across several papers, the evaluation metrics vary (EER, accuracy, PSR, AROC), limiting cross-study comparability. For example, Savvides et al. [25] use Peak-to-Sidelobe Ratio (PSR), reporting 100%, whereas another study measures the accuracy across AR, BERC, ORL, FERET, and Sheffield datasets, having EER ranging from 1 to 5% under some specific token conditions and 7 to 15% under shared or stolen token scenarios. Similarly, Faragallah et al. [27] and S.P. and Thomas [30] report EER values of 0% and between 1.38% and 1.76%, respectively, in contrast to Xu et al. [28], where accuracy ranges between 98.85% and 99.00%, while Alhumyani et al. [29] leverage the Area Under the ROC Curve (AROC) of 0.9951.

Among these modalities, iris-based feature extraction methods exhibit strong, stable performance, although perfect accuracy has not yet been achieved across all datasets. The accuracy varies, depending mostly on the transformations and dataset conditions. Some studies, such as Zuo et al. [32], evaluate cancellable iris transformations using ROC analysis, demonstrating that GRAY-COMBO and GRAY-SALT achieve low false-reject rates. At the same time, BIN-COMBO degrades under noise. In contrast, Rathgeb et al. [33] and Umer et al. [34] provide evaluations based on EER and CRR metrics, having values between 1.14% and 1.49% on the CASIA dataset and CRR = 100% and EER ≈ 0% for MMU1, UPOL, CASIA-v3, IITD, and UBIRIS datasets, respectively. Umer et al. [34] also report CRR = 99.83% and EER = 0.43% for the CASIA-syn dataset.

It is observed that deep learning improves discriminative capability; however, performance varies significantly across different modalities and fusion strategies. For example, for face modalities, as presented by Jang et al. [35], the EER values ranged from 0.0048% and 0.0961%. The accuracy values reported by Kim and Pyun [36] for ECG-based features ranged from 99.73% to 100%, and for the same modality, by Sakr et al. [40], ranged from 98.60% to 98.85%. Multimodal systems also exhibit different accuracies, ranging from 97.38% reported in Abdellatef et al. [38] to an EER of 6.67% reported by Kim et al. [41]. Further variability in performance and evaluation is demonstrated by unsupervised approaches, where Tarek et al. [42] report EER = 0.656% and accuracy = 99.35%, while Sardar et al. [43] report CRR = 100%, indicating that these results are dataset-dependent and may not generalise across different acquisition conditions.

For reliable polynomial reconstruction (recovering the polynomial from matched points) in fuzzy vaults, feature representations with lower EER and high intra-class consistency are more suitable. When selecting features for vault generation and construction, performance variability across datasets must be taken into account. Although fingerprint and iris modalities generally provide stable verification performance across multiple studies, the lack of uniformity introduced by the heterogeneity of evaluation metrics across the surveyed works, including EER, FAR/FRR, accuracy, CRR, AROC, and PSR, must be considered when integrating such features into fuzzy vault constructions. In particular, deep learning models exhibit a trade-off between discriminative capability and vault compatibility, often requiring transformation into fixed-length or quantised representations before integration, which may introduce additional reconstruction error.

#### 2.1.2. Encryption Phase (Vault Generation)

- Input to the Vault (*P*): For the generation of the vault, we require an input. These inputs are the actual, genuine data points for the individuals and represent a set of elements that capture biometric features. The consideration is that the input must be an unordered set of numbers that can be used to generate the vault. The input can be a numerical feature vector, derived from iris images (here) using a CNN model. The input can also be in the form of binary feature codes, a set of key-points, and scalar or quantised values [50]. The generalised input representation can be given as
  (1) $$P=\left\{p1,\;p2,\;p3\;\ldots pn\right\}$$
  where *p* represents elements of the set *P*, with x-coordinates taken from P and y-coordinates computed as F(x).
- Generating the Secret (S): The secret key is randomly generated and can be either in binary or numeric form. The secret S is divided into parts and distributed across the polynomial, where the construction of the coefficients of the polynomial is derived from the set S. For a polynomial having degree d, then S will be split into d + 1 parts [51].
  Using the coefficients of S, a polynomial F(x) of degree d is constructed.
  For the computation of the y-coordinate, evaluate the polynomial F(x) at each genuine data point x from P.
  The resultant (x,y) points lie on the polynomial constructed.
  (2) $$G=\left\{\left(x1,\;y1\right),\;\left(x2,\;y2\right),\;\left(x3,\;y3\right),\;\ldots .\left(xn,\;yn\right)\right\}$$
  *G* is the set of genuine points that lie on the constructed polynomial.
- Adding Chaff Points: These are random points that are added with the genuine set of points to conceal the vault and secure the data points generated by polynomial construction. These chaff points do not lie on the polynomial [50].

(3) $$C=\{\left(c1,\;c2\right),\left(c3,\;c4\right),\left(c5,\;c6\right),\;\ldots ..(cn,\;c_{n+1})\}$$

Combining a set of genuine points G and chaff points *C* forms the fuzzy vault.

(4) $$G\;\cup C=\left\{\left(x1,\;y1\right),\;\left(x2,\;y2\right),\;\left(x3,\;y3\right),\;\ldots .\left(xn,\;yn\right)\;\cup \;\left(c1,\;c2\right),\left(c3,\;c4\right),\left(c5,\;c6\right),\;\ldots ..(cn,\;c_{n+1})\right\}$$

(5) $$FV=\{\left(x1,y1\right),\left(x2,\;y2\right),\left(x3,\;y3\right),\;\ldots .\left(xn,\;yn\right),\left(c1c2\right),\left(c3,\;c4\right),\left(c5,c6\right),\;\ldots .(cn,cn+1)\}\;$$

The vault’s secret key S controls the degree of the polynomial. To handle the variations in the input, the Reed–Solomon code [14] is mostly used with the polynomial as follows:

RS encoding adds redundancy with the secret key S to correct errors if any occur. It is useful when the query input contains noise or when values are missing. Then, the secret key S can also be recovered, since the polynomial can be effectively computed.

If only error detection is required, a cyclic redundancy check (CRC) can also be implemented in place of RS.

#### 2.1.3. Decryption (Unlocking the Vault)

For the unlocking of the vault, a query set Q, consisting of the biometric feature vector of the individual, is given to the algorithm. The points in the query set should significantly overlap the points in P so that the vault will be unlocked. The decryption mechanism consists of four steps: first, the input is provided; then, the polynomial is reconstructed; next, the mechanism inspects errors; and finally, the key is matched [14].

- **Input during Unlocking**: The Q set of query points is taken as input. Also, the vault generated during the encryption process consists of the genuine set of points G and a set of random points C, which are also taken as input for the decryption process.
- **Matching the Points**: For each data point in q ∈ Q, if the point matches the x-coordinate of any point in the vault. The obtained set may contain both the genuine points and the random chaff points, where the random points are ignored during the polynomial reconstruction [51].
- **Polynomial Reconstruction**: Unlocking the vault requires the reconstruction of the polynomial *p*, which is of degree *d*, as the secret key can be retrieved only by obtaining the coefficients of the reconstructed polynomial. For reconstruction of a degree polynomial, *d* + 1 distinct projections or points are required. Combinations of these d +1 points are selected and are used to construct a Lagrange’s interpolating polynomial. The coefficients obtained from this polynomial are then mapped back to the decrypted key S*, using the same decryption algorithm used to encrypt S. The reconstructed secret S is obtained.
- **Error Checking**: A CRC is employed to check the accuracy of the obtained coefficients and verify the integrity of the decrypted key, S*. The error-checking mechanism does not disclose any information and is only employed to check the errors [52]. This prevents external attacks, as the attacker cannot distinguish between correct and incorrect projections because the real and fake points are not differentiable.

**Figure 4:**
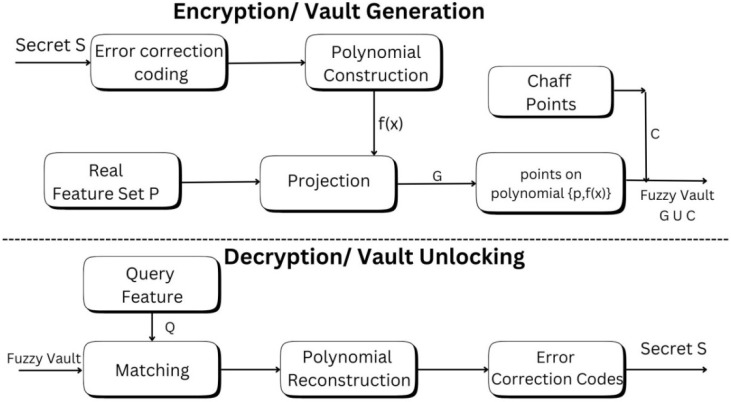
Encryption and decryption of fuzzy vault.

For the accurate decoding of the secret key, the list of query points must overlap with the template feature set P for at least d + 1 points. The correct combination of the points from Q and P generates the secret key and ensures that it matches the template features. The correct secret key is decoded by the overlap between query and template biometrics when they belong to the same person. The decoded key matches the original key that is stored in the database, and the decoding is successful if the keys are similar. The encryption and decryption process of the fuzzy vault is presented in Figure 4.

- **Lagrange Interpolation for Polynomial Reconstruction**: It is a method that is utilised in the reconstruction of the polynomial, as we are not aware of what the function F(x) is, but we have the points (x, y). Using Lagrange interpolation, we can compute the function F(x) in the context of the fuzzy vault scheme by obtaining the matching points from the vault. If the polynomial is reconstructed correctly, its coefficients form the secret S, completing the authentication process [51].

#### 2.1.4. Algorithm 1: Fuzzy Vault Encryption (Vault Generation)

Algorithm 1 outlines the step-by-step procedure for fuzzy vault encryption, also referred to as vault generation. In this process, a biometric feature set P is extracted from biometric samples such as iris or fingerprint data. A random secret S is generated and encoded into a polynomial F(x) of degree d. The polynomial is evaluated at each biometric point to produce genuine points G, while chaff points C are randomly generated to obscure the genuine points. Finally, the genuine and chaff points are combined into a single unordered set to form the fuzzy vault FV.

**Algorithm 1:** Fuzzy vault encryption (vault generation)**Input:** Biometric feature set P = {p1, p2, …, pn} extracted from biometric samples (e.g., iris, fingerprint) **Output:** Fuzzy Vault FV, Secret S
    1.Input biometric features (P): Use a feature vector extracted from biometric data.    2.Generate secret (S): Create a random key to be protected S.    3.Construct polynomial (F(x)): Encode the secret S into a polynomial F(x) of degree d.    4.Create genuine points (G): Evaluate polynomial F(x) at each point x € P to get (x, y) pairs.    5.Generate chaff points (C): Generate random points that are not on the polynomial.    6.Vault formation (FV): Combine sets G and C into a single unordered set. F = GUC


#### 2.1.5. Algorithm 2: Fuzzy Vault Decryption (Vault Unlocking)

Algorithm 2 outlines the step-by-step procedure for fuzzy vault decryption, also referred to as vault unlocking. In this process, a query biometric feature vector Q is obtained from the user seeking authentication and matched against the stored fuzzy vault FV. The points in Q corresponding to FV are identified, and Lagrange interpolation is applied using a minimum of (d + 1) matching points to reconstruct the polynomial. The secret S is then recovered from the reconstructed polynomial coefficients. The recovered secret is verified using CRC or Reed-Solomon decoding, and based on the verification result, the system either grants successful authentication or returns an authentication failure.

**Algorithm 2:** Fuzzy vault decryption (vault unlocking)**Input:** Query biometric feature vector Q, Fuzzy Vault FV **Output:** Reconstructed secret S* (or authentication failure)
    1.Input query features (Q): Obtain features from the user seeking authentication.    2.Correspondence points: Determine the points in Q corresponding to FV.    3.Rebuild polynomial: Employ (d + 1) match points and Lagrange interpolation.    4.Recover secret (S*): Retrieve secret from reconstructed polynomial coefficients.    5.Secret verification: Check with CRC or Reed–Solomon decoding.    6.Decision: Successful verification leads to successful authentication or failed authentication, respectively.


### 2.2. Implementation and Applications of Fuzzy Vault in Biometric Authentication Systems

Fuzzy vaults’ tolerance to naturally occurring biometric variations and to variations caused by differences in image exposure has led to extensive use of this mechanism across various biometric cryptosystems. Also, it provides advanced security as it does not store the raw data directly in the database; instead, it stores the auxiliary data (stored helper data combining encrypted template and key) that includes a set of points comprising both genuine points and the arbitrary chaff points, making it challenging for intruders to deduce the biometric templates even if they gain access to the database. This mechanism has been efficiently implemented across various biometric recognition systems and thus makes it ideal to use for even multimodal biometric authentication systems. With the advent of new technologies like AI and ML, this mechanism is being combined with these emerging innovations to enhance its performance.

#### 2.2.1. Fuzzy Vaults in Biometric Cryptosystems Using Fingerprint Dataset

Shenglin Yang and Ingrid M. Verbauwhede [53] first implemented the fuzzy vault scheme on a fingerprint dataset to generate a secure fingerprint recognition system. They also incorporated an adaptive alignment mechanism to align the data points, then extracted shift-invariant features. The dataset they used consisted of 100 images, with each finger having 10 images. For accurate matching, three images were used as query templates. Along with the original data points, 200 imposter data points were added, and, in doing so, the performance rate of this procedure was estimated at 83%.

Umut Uludag, Sharath Pankanti, and Anil K. Jain [54] used the fuzzy vault mechanism on the IBM-GTDB fingerprint dataset, consisting of 100 images of manually marked fingerprint minutiae, and, to reduce variations, incorporated Galois fields and quantisation techniques. Cyclic redundancy checks were implemented to detect errors. The system achieved a Genuine Acceptance Rate (GAR) of 79% and a False Acceptance Rate (FAR) of 0%, with no unauthorised users being recognised as legitimate.

Karthik Nandakumar, Anil K. Jain, and Sharath Pankanti [55] proposed a fully automatic fuzzy vault system, utilising ridge endings and bifurcations to extract the most prominent features of the fingerprint and, for data alignment, high-curvature points. To handle fingerprint distortions, an adjusting bounding box was also incorporated, along with a trimmed Iterative Closest Point (IPC) algorithm that, unlike the regular IPC, discarded the worst matches (i.e., outliers). The system was evaluated on two datasets, FVC2002-DB2 and MSU-DBI, achieving GARs of 91% and 82.5%, respectively.

V. Evelyn Brindha [56] proposed a secure multimodal biometric authentication system that utilised the Fingerprint Verification Competition (FVC) fingerprint dataset as well as the palm print dataset for enhancing the performance of the authentication system in recognising only the legitimate users. To protect the template, fuzzy vaults were implemented, thereby enhancing the authentication system’s security.

Wencheng Yang, Jiankun Hu, and Song Wang [57] proposed a novel method for securing the biometric template using the Delaunay triangle-based fuzzy vault. The proposed system supported cancellability, that is, the ability to replace compromised data points. The fingerprint images obtained from the FVC2002 DB1 and DB2 datasets were analysed. Each fingerprint, based on minutiae points, was divided into a triangle, and, to make the templates non-reversible, polar transformations were applied. The performance in terms of Equal Error Rate (EER) was estimated to increase after applying the transformation, from 5.54% (DB1) and 3.4% (DB2) to 8.46% (DB1) and 5.7% (DB2). The security of the system is primarily governed by the number of minutiae triangles (TN), chaff points (cfN), and the polynomial degree used in the fuzzy vault.

Reza Mehmood and Arvind Selwal [58] further enhanced fuzzy vaults by implementing polynomial transformations, making it even harder to recover the secret key. To increase the degree of the polynomial, an integral function transformation was applied to the key, which was stored as polynomial coefficients, thereby rendering its reconstruction complex. The system was trained on the FVC2002-DB1, DB2, DB3, and DB4 (Set B) datasets, each consisting of 80 fingerprint images from 10 users. The Genuine Acceptance Rates (GARs) were 92% for Polynomial Degree 3, 90% for Polynomial Degree 4, and 85% for Polynomial Degree 5. The paper emphasised the Security–Accuracy trade-off: increasing the polynomial degree increased security and reduced the False Acceptance Rate (FAR), but it also increased the False Reject Rate (FRR), i.e., denying access to legitimate users, thereby affecting convenience for legitimate users.

Vivek Singh Baghel, Surya Prakash, and Ity Agrawal [59] introduced an enhanced method of minutiae extraction mechanism involving the concept of Principal Component Analysis (PCA) for the proper alignment of the templates, eliminating the extra storage requirement for auxiliary data. For faster decoding of the vault, only the well-separated minutiae points, which were near the singular point, were considered during authentication. The system was trained on the standard datasets FVC2002 DB1, FVC2002 DB2, and FVC2004 DB, and the Genuine Acceptance Rate (GAR) was computed to be higher than that of traditional methods. This mechanism provided stronger security and faster authentication.

Prabhjot Kaur and Nitin Kumar [60] introduced the concept of deep learning in biometric cryptography for efficient feature extraction. They combined a convolutional neural network, which extracted prominent features from the FVC2004 Fingerprint Database (DB1, DB2, DB3, DB4), consisting of 1440 fingerprint images from 12 subjects, and the fuzzy vault scheme for efficient biometric template protection. The proposed method outperformed the conventional method, achieving 89.9% accuracy. In terms of statistics, the Peak Signal-to-Noise Ratio (PSNR), a statistical method for evaluating the quality of extracted biometric features, was computed at 9.8762 on DB4, confirming that the extracted features retained sufficient information for successful decryption.

#### 2.2.2. Fuzzy Vaults in Biometric Cryptosystems Using the Iris Dataset

The iris is a prominent biometric feature because it is unique to each individual and accurately authenticates identity. Youn Joo Lee [61] proposed a novel method of implementing the fuzzy vaults on the iris datasets, aiming for better key management and securing the iris biometric templates simultaneously. Independent Component Analysis was encompassed in the feature extraction method, as this method proved to perform similarly to Daugman’s method, extracting multiple local iris feature vectors, unlike global iris codes. A pattern classification method, specifically K-means clustering, was applied to handle the variations in the sixteen 27-bit binary feature vectors. They then combined these feature vectors with a 128-bit AES-generated key and applied Reed–Solomon codes for error correction and Lagrange’s interpolation method for polynomial regeneration. The system was tested and trained on the BERC Iris database, consisting of 990 iris images of 99 individuals with a resolution of 640 × 480, where the False Rejection Rate (FRR) was computed as 0.775% at 0% False Acceptance Rate (FAR), and the Genuine Acceptance Rate (GAR) was 99.225%.

Youn Joo Lee [13] enhanced this method by implementing a shift-matching technique in which the distance between the iris feature prototype and the test iris image feature vectors was measured using horizontal shifts in the ROI, thereby solving the problem of feature alignment without pre-alignment techniques. Iris Pseudo Codes (IPCs) converted the extracted features, represented as clusters, into finite field elements for vault encoding. The BERC, CASIA V1.0, and CASIA-IrisV3-Interval datasets were used to implement the system, achieving Genuine Acceptance Rates (GARs) of 99.675% for BERC, 89.78% for CASIA V1.0, and 92.66% for CASIA-IrisV3-Interval at a 0% False Acceptance Rate (FAR).

Further, Arzoo Arora and Prof. Ravindra Divekar [62] implemented the fuzzy vault mechanism for securing biometric templates on iris and fingerprint datasets, utilising a 2D Gabor Wavelet Filter for 256-bit feature vector extraction and Hamming and Euclidean distances for template matching. The system was evaluated on the CASIA iris database, achieving a False Acceptance Rate (FAR) of 0.01% and False Rejection Rate (FRR) of 0.15%.

Christian Rathgeb developed a multi-biometric fuzzy vault [63] that aimed at securing the biometric feature templates of both eyes of an individual in a single vault. They named it feature-level fusion (combining features from multiple modalities into a single representation), where the upper and lower sections of the iris images were discarded, and only the most prominent features were combined from both eyes. The iris feature codes were then converted to unordered feature sets by dividing them into blocks using the Binary-to-Integer Ordered-to-Unordered Transformation. The proposed mechanism also protected the system against cross-matching attacks by incorporating Record-Specific Public Bijection, a public transformation function initialised with the hash of the secret polynomial. The system was trained on the CASIAv3-Interval database for left-eye images and the IITDv1 database for left- and right-eye images for testing.

Tong-Yuen Chai and Bok-Min Goi [64] proposed an alignment-free cancellable iris key binding, without using error correcting codes for key retrieval. They used a Bloom filter to extract iris image features from the CASIA v3-Interval iris database, which consists of 124 classes and 86 left-eye images. The technique works by dividing the iris codes into blocks and applying the filter to each block, ensuring alignment-free encoding and reducing the impact of head rotation. To strengthen non-invertibility and reduce security leakage, Indexing First One (IFO) Hashing and Modulo thresholding were implemented. The method’s performance was evaluated using the New Key Retrieval Rate (KRR) metric, which directly quantifies key retrieval success under genuine queries, achieving an optimal genuine acceptance rate (GAR) of 96.37% with zero FAR at a hashed code length of 100.

Katarzyna Bobkowska, Khaled Nagaty, and Marek Przyborski [65] developed a centralised security framework utilising multimodal biometrics, including face, fingerprint, and iris data. They included biometric mapping schemes, LSB steganography, and fingerprint fuzzy vault to strengthen security for e-passports. Biometric mapping schemes include single, dual, and distributive, where single mapping produces a location map, dual mapping generates a concealed single image from two biometric images called a biostego image, and distributive mapping divides a single biometric image, providing security through data distortion. To reinforce security and conceal the encoded location map, LSB steganography (Least Significant Bit) was implemented, along with a fuzzy vault, using a fingerprint dataset and a third-degree polynomial to encode the location map. For face detection, the Viola–Jones algorithm was implemented, and Histogram stretching was used to improve the biostego image representation. The system was trained on a Coloured passport face images (real e-passport images) dataset; Coloured iris images from the Karolinska Directed Emotional Faces (KDEF) database, which is a freely available dataset; and grey fingerprint images obtained from an online open-source database. Structural Similarity Index (SSI), Peak-Signal-to-Noise-Ratio (PSNR), and 2D Correlation Coefficient (r) are the statistical methods used to compute the similarity between original and decoded biometric images.

Mohammed A. Taha and Hanaa M. Ahmed [66] presented a grey-level co-occurrence matrix (GLCM)-based texture extraction mechanism, along with multi-key encryption, to further enhance the security of fuzzy vaults. To avoid brute-force attacks, the search space was expanded by using two keys: one for encrypting the biometric template and another for encrypting the vault. The proposed mechanism was trained and tested on the CASIA v1 database, consisting of 756 iris images from 108 users, and the IITD v1 dataset, comprising 2240 iris images from 224 users, with a Genuine Acceptance Rate (GAR) of 99.86% for the CASIA v1 dataset and 99.88% for the IITD v1 dataset.

#### 2.2.3. Fuzzy Vaults Implemented Using Other Modalities

Fuzzy vaults have been implemented across various modalities for secure storage of biometric templates. The authentication system using ear biometrics, Gabor filters, and fuzzy vaults to extract and secure the biometric feature has achieved an accuracy of 98.83%. To minimise the dimensionality of the feature vectors obtained from the EAR-IIT Delhi dataset version 1.0, Multi-Manifold Discriminant Analysis (MMDA) is employed [67].

Another proposed implementation of the fuzzy vault [68] is based on the AR face dataset, with the dual-key concept introduced into the fuzzy system. One key is generated from the features of the face and iris of the operator using a confidence interval, and the second key, which is used to generate the polynomial, is based on memory retention of the operator. To compress the feature dimensionality, Principal Component Analysis (PCA) is applied to the face feature vector. For vault sizes ranging from 100 to 1000, encryption and decryption took at most 3.5ms.

With the incorporation of the concepts of machine learning, the features, i.e., frequency-weighted power (FWP) features from EEG data and minutiae from fingerprints, are extracted using a deep convolutional neural network (CNN), which is employed along with the CNN classifier-based score fusion algorithm. It uses a publicly available electroencephalogram (EEG) dataset comprising 28 participants with 19 channels. For fingerprint data, it utilises the FVC2004 dataset, consisting of 80 fingerprint samples from 28 persons. The performance of the proposed system is computed as 0.25% Equal Error Rate (EER) for EEG and 0.20% for fingerprint in the unimodal setup, and 0.10% in the multimodal setup [69].

As noted in Table 2, fingerprint-based fuzzy vaults have been comprehensively investigated across databases such as FVC2002, FVC2004, and IBM-GTDB. Adaptive alignment-based algorithms [53], Galois fields-based algorithms [54], and trimmed ICP-based algorithms [55] have reported GARs between 79% to 91%, and 0% FAR for others. Recently developed algorithms in CNN-based extraction of fingerprint features [60] reported an accuracy of 89.9% with a PSNR of 9.8762, demonstrating the ability of deep learning algorithms to preserve important information for decryption.

In iris-based vaults, several studies have consistently demonstrated high performance, with GARs above 99%, using approaches such as ICA and AES-based binding [61], shift-matching [13], and multi-key encryption [66]. Of special interest is the fact that approaches without error-correcting codes, using Bloom filters [64], achieved a GAR of 96.37% with 0% FAR, proving the efficacy of alignment-free encoding mechanisms.

Multimodal and hybrid approaches, combined with EEG, facial, and palmprint modalities, are more robust, particularly when used with deep learning and score fusion techniques [69]. Multimodal configurations have been shown to achieve EERs as low as 0.10%, demonstrating the advantage of combining modalities for improved recognition accuracy at no error cost.

#### 2.2.4. Recent Advances in Fuzzy Vault-Based Systems

A single-factor scheme designed to mitigate statistical biases was proposed in a recent study where fuzzy vaults with Reed–Solomon error correction and Peterson–Berlekamp–Massey decoding algorithms, along with a quartile-based encoding strategy, were used [70], and were implemented to reduce statistical leakage, achieving False Acceptance Rates (FARs) of 1.3% for fingerprints, 2% for ECGs, and 0% for faces. Despite a lower False Acceptance Rate, the findings suggest a trade-off between usability and resistance to statistical attacks evaluated on datasets such as FVC2002, PTB, and LFW, with False Rejection Rates (FRRs) of approximately 56% for fingerprints and 75% for facial data under certain configurations.

A study explores the integration of fuzzy vaults with blockchain-based decentralised architectures [71] to overcome limitations in centralised storage and data integrity. By storing biometric tokens in the InterPlanetary File System (IPFS) and indexing them on an Ethereum-based blockchain, the framework combines asymmetric cryptography with distributed ledger technology. The research applies feature extraction techniques, including MRG and GLCM for the dorsal hand images and HOG based on deep learning models for facial feature recognition, which yields an impressive result in terms of authentication accuracy, with a high accuracy rate of almost 99.8% and EER close to 0.08 in the CASIA-FaceV5 and 11 k Hands databases. Nevertheless, the capacity of blockchain technology is constrained, and, due to transaction delays, scaling becomes a challenge in large-scale implementations.

Enhanced fuzzy vaults have been proposed wherein integration of elliptic curve cryptography (ECC) with biometric template protection is leveraged in [72]. The feature extraction is carried out using a commercial SDK; the features are then quantised and mapped to elliptic curve points, allowing for secure key binding independent of conventional error correction codes. The reported Equal Error Rates (EERs) are as low as approximately 0.005% on certain FVC2002 datasets and around 2.4% on FVC2004. While the study guarantees unlikability, revocability, and non-invertibility, it does not address sensor-level spoofing attacks, and under conditions involving significant finger displacement or rotation, performance degradation is observed.

Voice-based biometrics have also been used to generate secure keys, leveraging neural fuzzy extractor frameworks in [73]. In this study, VGG19-based convolutional autoencoders, in combination with trigonometric correlation function-based hybrid neural architectures, have been incorporated to extract features from Short-Time Fourier Transform (STFT) representations of speech signals. While achieving an EER of approximately 2.1% on a custom dataset and 3.2% on a public dataset, the system is sensitive to external noise. Also, the study suggests that increasing network complexity may introduce instability and does not consistently improve performance.

A multi-layered framework combining a fuzzy vault and a lightweight variant of the Advanced Encryption Standard (AES) is proposed to strengthen security in resource-constrained environments [74]. The study achieves FAR and FRR values of approximately 1.1% and 1.5%, respectively, for minutiae-based fingerprint features and Gabor-filter-based iris features. While maintaining a reduced computational overhead, the paper addresses the inherent challenge of balancing protection with error tolerance required for biometric variability.

Another study in the domain of securing biometric templates, combining both classical and post-quantum cryptographic settings, is presented in [75]. It combines the fuzzy vault scheme with Oblivious Pseudo-Random Functions (OPRFs) and Key Encapsulation Mechanisms (KEMs), achieving False Match Rates (FMRs) of 1.13% on the CASIA-FPV5 database and 1.01% on the FVC2004 DB-1 dataset. Although the research demonstrated a 99.9% cryptographic success rate for post-quantum lattice-based (OPRF), the approach highlights increased computational and communication overhead in lattice-based instantiations and potential limitations in more adversarial settings.

The authors in [76] propose an Oblivious Key-Value Store (OKVS) protocol to address two main limitations in traditional fuzzy vault systems: cross-matching vulnerability and excessive storage overhead. The study combines multimodal fingerprint minutiae features with deep learning-based facial embeddings, using quantisation techniques in which the system treats the biometric features as “keys” and the Multi-Threshold Secret Sharing (MTSS) secret shares as the corresponding “values”. The experimental results achieved a False Acceptance Rate (FAR) of 0.00%, a False Rejection Rate (FRR) of 4.00%, and an EER of 3.00%. The study shows a trade-off between efficiency and performance: improving matching accuracy by increasing quantisation precision increases storage requirements and computational overhead.

Yet another study addresses the high computational intensity and heavy resource consumption of traditional fingerprint fuzzy vaults by presenting a square-boundaries-based chaff generation method in [77] to increase the difficulty of distinguishing genuine and chaff points. It incorporates spatial and angular relationships on fingerprint minutiae, and results indicate a trade-off between accuracy and computational efficiency, achieving a False Rejection Rate (FRR) of approximately 15.3% and a False Acceptance Rate (FAR) of 8.6% with relatively low processing times, but demonstrated a higher computational cost with more accurate configurations. The paper demonstrates the importance of chaff point design in balancing security strength and system efficiency.

#### 2.2.5. Design Considerations, Security–Accuracy Trade-Offs, and Emerging Trends in Robust System Modelling

Some key parameters, like feature representation, polynomial degree, and chaff point configuration, shape the design of the fuzzy vault biometric system. These parameters also influence security, accuracy, and computational complexity. Careful tuning of these parameters is critical to balance accuracy, robustness, and security of these authentication systems. The choice of feature extraction methods affects the system’s robustness: more modern approaches offer improved resilience to noise and misalignment compared to traditional approaches. Still, these techniques often come with high computational cost, alignment sensitivity, and scalability limitations. Thus, recent trends focus on integrating deep learning, multimodal biometrics, and advanced cryptography to enhance performance and security, and on providing hybrid solutions to improve the security of biometric authentication systems, especially fuzzy vaults. A security–usability trade-off is present, as processing overhead and increased False Rejection Rates can be seen in stronger cryptographic settings. The following section discusses these parameters in more detail, and Table 3 summarises the analytical observations.

- Design Parameters:

A comparative analysis of the surveyed fuzzy vault systems reveals that transformed or learned representations, such as Delaunay triangle groupings [57,60] and CNN-based embeddings, improve robustness and intra-class variations in contrast to traditional techniques of feature extraction like minutiae-based representations [54,55], which are sensitive to alignment and noise but do offer high discriminative capability. Along with feature representation, system performance is strongly governed by polynomial degree and chaff point configuration, where lower-degree configurations yielded higher acceptance rates [58] and higher security, but at the cost of increased rejection rates for genuine users. The security performance was significantly improved with the introduction of large chaff point sets, but this also introduced substantial computational overhead.

- Security–Accuracy Trade-Offs:

Trade-offs between security, accuracy, and usability are consistently observed across the literature, even in contemporary fuzzy vaults. As reported in [57], resistance against brute-force attacks and polynomial reconstruction increases with polynomial degree, but this also increases the False Rejection Rate (FRR), here over 40%, due to the need to correctly match a larger number of genuine points. Conversely, a drop in Genuine Acceptance Rates (GARs) is observed in [58] as the polynomial degree increases. These trade-offs can also be observed in recent work, where resistance to distribution-based attacks is mitigated by incorporating statistical bias techniques into single-factor fuzzy vaults, but this results in substantially higher False Rejection Rates, affecting usability [70]. Similarly, multi-layered encryption frameworks [74] do improve efficiency and energy consumption for edge environments, but require balancing between biometric error tolerance and cryptographic strength. It is also observed that increasing the number of chaff points increases resistance to correlation and brute-force attacks in [54] and [55], but this is accompanied by increased runtime and decoding complexity.

- Robustness and Feature Design:

Feature design and preprocessing strategies greatly define the extent of robustness to noise, distortion, and intra-class variability. Additional preprocessing or even helper data is required in a raw coordinate-based system, as seen in early implementations such as [54], whereas, in contrast, deep learning-based approaches [60] learn invariant representations, thereby further enhancing robustness. However, this method introduces additional computational cost and dependency on training data. Improved tolerance to rotation and translation, and reduced alignment errors, can be observed in alignment-free or invariant representations [53,57] and in local shift-matching techniques in iris-based systems [61], respectively.

- Parameter-Level Insights:

The trade-off between security and error tolerance is directly determined by the polynomial degree, while the chaff point density determines the level of obfuscation and computational complexity. Recent systems, such as OKVS-based and neural fuzzy extractors [76], aim to relax the constraints imposed by polynomial degree and chaff density, but, in doing so, introduce alternative dependencies on feature quantisation, model stability, and computational overhead. Efficient encoding is evident in fixed-length feature representations, such as CNN-derived vectors [60], whereas, despite preserving structural information, alignment handling is required in variable-length representations, such as minutiae sets [55]. Apart from polynomial degree, chaff density, and feature representations, additional factors, such as decoding algorithms, also influence system efficiency and scalability. Careful tuning of these parameters is required to achieve an optimal system design that balances accuracy, security, and computational feasibility.

- Common Limitations:

Achieving both high security and practical usability in fuzzy vault systems is difficult, as evidenced by high False Rejection Rates, particularly in high-security configurations or noisy environments [57,58]. In systems with large chaff sets, computational complexity becomes another challenge, as decoding time in early settings reached 50 s [54]. At the same time, later optimisations reduced it to a few seconds through feature filtering techniques [55]. The use of auxiliary data introduces potential vulnerabilities, and alignment dependency continues to affect many fingerprint-based systems.

- Emerging trends:

A shift towards hybridisation is clearly seen, where, to overcome traditional limitations, deep learning, multimodal biometrics, and cryptographic techniques are combined. To improve robustness and security, deep learning-based systems [60,70] leverage learned feature representations, and resistance to spoofing is improved by multimodal and EEG-based approaches [56,69]. Blockchain-integrated frameworks and cryptographic primitives, including elliptic curve cryptography and post-quantum primitives, reinforce security and decentralisation. However, these techniques introduce computational overhead, storage challenges, and scalability constraints, underscoring the need for lightweight designs.

#### 2.2.6. Practical Deployment Considerations in Fuzzy Vault-Based Systems

The practical deployment of fuzzy vault-based biometric systems introduces several challenges beyond theoretical design and performance evaluation; these include computational constraints, scalability, user experience, and environmental stability. In resource-limited environments, such as mobile and edge devices, a careful assessment of computational constraints is required. Execution times of approximately 20 s on constrained platforms such as the Raspberry Pi [78] have been reported for polynomial reconstruction, with quadratic complexity [79], imposing significant computational overhead and contributing to high energy and processing costs. As discussed, increased chaff density increases false-match probabilities; consequently, large chaff point sets are required for security, which, in turn, degrades decoding efficiency [78].

In large-scale systems, maintaining acceptable response times is essential; scalability is another critical challenge in practical deployments. Direct retrieval of individual fuzzy vaults is enabled by combining cloud-based storage (Azure Cosmos DB) with user-specific identifiers [78], avoiding exhaustive database searches. The system’s usability and acceptance are adversely affected by high False Rejection Rates. Although some systems achieve an acceptable FRR, there are reports of high False Non-Match Rates (FNMRs) of up to 18% [78] under high-security settings, thereby making user experience a key consideration. Similarly, ref. [80] attributes frequent authentication failures to an imbalance between false and genuine matches, which hinders successful polynomial reconstruction.

Substantial discrepancies between enrolment and query data, introduced by variations such as global rotations, translations, and non-linear deformations, significantly undermine the system’s reliability [80]. Environmental variability, such as inconsistent biometric measurements affected by noise and acquired from different devices, may pose serious credibility issues for authentication systems. Integrating the use of commercial off-the-shelf sensors, application-layer interfaces, and cloud-based services [78] is considered to be feasible, but may introduce performance degradation arising from feature transformation losses [81].

## 3. Challenges and Open Issues

Despite the prevalent use of fuzzy vaults in securing biometric templates, there are still some implementation problems across modalities:

- Alignment Dependency: Reliable alignment of biometric traits continues to pose the biggest problem to fuzzy vault-based systems, most notably to the fingerprint and iris modalities. Various alternative solutions, like shift-matching [13] and adaptive alignment [53], have been offered, but the pre-alignment or shift-tolerance coding requirement still impacts system development and increases computational expenses.
- Security–Usability Trade-off: As remarked by Reza Mehmood and Arvind Selwal [58], increasing the polynomial degree enhances security by lowering the False Acceptance Rate (FAR). Still, at the cost of increasing the False Rejection Rate (FRR), it prevents authorised users from accessing the system. This trade-off sacrifices the system’s usability and convenience.
- Biometric Variability: Random variation in biometric samples by noise, exposure to images, or time variance affects the consistency of vault unlocking. The problem is addressed in Section 2.2, where tolerance to such variability is cited as one of the fuzzy vault’s strongest benefits, yet also as a technical challenge to be addressed by error-resilient handling schemes.
- Computational Overhead: The decryption and encryption operations carried out in fuzzy vault schemes demand polynomial calculations, chaff point addition, and Lagrange interpolation. These tasks, especially when combined with deep learning-based feature extraction methods, can impose higher computational burdens, as discussed in the last part of this manuscript.
- Template Security Threats: Although fuzzy vaults circumvent the storage of raw biometric templates, vulnerable auxiliary data (e.g., vault points) remains a threat. Section 1.2 states that vulnerable templates cannot be replaced, and that biometric data protection is generally overlooked due to myths about the inherent security of biometric systems.

### Evolving Attack Landscape on Fuzzy Vault-Based Biometric Cryptosystems

Biometric authentication systems are vulnerable to attacks at multiple stages, including data acquisition, feature extraction, template storage, and matching. While such threats affect biometric systems broadly, fuzzy vault-based cryptosystems introduce additional vulnerabilities due to their polynomial encoding and chaff-based obfuscation mechanisms.

**Traditional Access Attacks:** Fuzzy vault systems are susceptible to several traditional access attacks. In intrusion attacks, because fuzzy vaults must tolerate a threshold of dissimilarity to function properly, an attacker may use known information from one system to generate fake query data and gain unauthorised access to another system if the threshold is set too high. In combination attacks, by combining the legitimate biometric with its own data, an attacker may try to gain access to the same system or attempt to access other systems as well. In injection attacks, by directly injecting its own biometric data into the stored vault, an attacker may try to confuse the vault system into thinking that the genuine points are the attacker’s biometrics, thus making the actual genuine points behave as chaff points, which falsely reject the legitimate user and grant illegal access to the intruder [82].

**Brute-Force Attacks:** The finite nature of the vault makes the fuzzy vault biometric system vulnerable to brute-force attacks, especially during the vault formation and interpolation phase. This issue is addressed in [83] by combining SHAKE-128, Kyber-1024, and AES-256-CBC. The study highlights a security–performance trade-off whereby implementing high-level security mechanisms reduces the system’s recognition accuracy and usability.

**Correlation-Based Attack:** It is established that when two vaults are created using the same source (biometric data), the statistical correlation can reveal the genuine points hidden among the chaff points. The research [84] unlocked 94 of 100 targeted fuzzy vault pairs and concluded that, without additional security measures, fuzzy vault systems are highly susceptible to correlation attacks.

**AI-Driven Adversarial Attacks:** Recent advances in adversarial machine learning have exposed critical vulnerabilities in image-based recognition systems. Some noise, known as imperceptible perturbations, can be introduced to the biometric inputs using transferable adversarial example generation techniques. These perturbations mislead the recognition models by preserving visual realism. A similar technique, the dual-phase reversible adversarial framework (DP-TRAE), misleads the AI model and enables recovery of images with minimal degradation in quality [85]. Such perturbations, when applied to feature templates, may cause incorrect polynomial reconstruction and key retrieval failures, resulting in high FRR in authentication systems. This emphasises the necessity of incorporating robust feature extraction and verification resilient to adversarial attacks.

**Advanced Data Hiding and Steganographic Threats:** Emerging breakthroughs in deep steganography pose a potential threat to biometric systems. Templates protected within biometric security constructs can be embedded with hidden information in a highly undetectable manner by leveraging channel-wise attention and global–local feature aggregation, selectively modifying texture-rich regions, thereby making detection extremely challenging. These hidden modifications, introduced using models like CAISFormer [86], may create inconsistencies in authentication systems by interfering with the feature extraction process. Subtle alterations in feature representations can compromise the reliability of biometric authentication systems, such as fuzzy vaults, and degrade their matching performance.

**Statistical Attacks on Fuzzy Vault Schemes:** To investigate vulnerabilities in fuzzy vaults, especially their susceptibility to statistical attacks, a recent work exploited the non-uniform distributions of stored biometric features to compromise the vault. The work simulated the attack using a Uniform Attacker (UA), a Partial Knowledge Attacker (PKA), and a Distribution-Knowledge Attacker (DKA), demonstrating that attackers with knowledge of the statistical distribution had a massive advantage and that the more specific the statistical knowledge, the easier it is to break the vault [70].

The inherent trade-off between security and system performance is the key challenge in defending against these biometric and cryptographic attacks.

These challenges underscore the need for further research to strengthen fuzzy vault constructs, balance security and usability, reduce computational overhead, and ensure strong protection against emerging attack models in biometric cryptosystems.

## 4. Future Direction

As biometric cryptosystems evolve, many future research directions aim to improve fuzzy vault implementations by enhancing strength, scalability, and confidentiality. In recent years, fuzzy vault systems have shifted toward more practical, privacy-conscious designs. One area that has gained increasing attention is the use of deep learning, especially CNNs, to improve biometric feature extraction. Rathgeb et al. [87], for example, developed a face-based fuzzy vault that transforms CNN outputs into integer-valued sets, and their results on FERET and FRGCv2 showed a false non-match rate under 1% at a false match rate of just 0.01%. This represents strong performance for a vault system using real-world data. At the same time, combining different biometric traits is also proving effective. Their later work [88] introduced a vault that fuses face and fingerprint features, reaching perfect recognition and over 30 bits of false accept security. Apart from that, researchers have begun moving away from alignment-heavy designs. Ali et al. [89] proposed a fingerprint scheme based on minutiae triplets that eliminates the need for pre-alignment, making it more flexible and easier to implement. Finally, privacy during matching is being tackled through homomorphic encryption. Pradel and Mitchell [90] proposed a setup in which matching occurs entirely on encrypted biometric data—so the system processes no raw biometric data directly. These directions show a clear move toward more usable, secure, and privacy-respecting fuzzy vault implementations.

Recent advances in privacy-preserving computation have introduced authenticated encrypted search mechanisms to support secure querying over encrypted cloud-stored data. One such scheme, constant trapdoor public-key authenticated encryption with keyword search (CT-PAEKS) [91], allows for users to retrieve encrypted biometric records without exposing either the search query or the underlying data. Built on lattice-based cryptography, CT-PAEKS resists both quantum-computing and internal keyword-guessing attacks while maintaining computational efficiency on mobile cloud platforms. When integrated with template protection schemes such as fuzzy vaults, this approach supports scalable biometric authentication in distributed environments where query privacy and data confidentiality must both be preserved.

Similarly, for secure and verifiable identity management, blockchain-based decentralised identity (DID) frameworks [92] have emerged, ensuring tamper-resistant storage and transparent verification of identity credentials by leveraging distributed ledger technology and cryptographic data structures such as Merkle trees, thereby making identity verification independent of centralised authorities. For large-scale applications, such as IoT ecosystems and cross-organisational authentication scenarios, fuzzy vault mechanisms can be integrated with blockchain-based identity systems to securely bind biometric templates to decentralised records, enabling trustless authentication and protecting templates from data breaches.

## 5. Conclusions

This survey provides a comprehensive evaluation of fuzzy vault-based biometric cryptosystems, emphasising the critical influence of feature representation, polynomial degree, and chaff point configuration on system performance. The research presents a comparative analysis of feature extraction methods and the modalities used in biometric authentication systems. Furthermore, it analyses the implementation of fuzzy vaults across different modalities and setups. The paper highlights key research gaps, including the need to construct low-FRR yet secure vaults and the lack of standardised benchmarking frameworks. Limitations in scalable blockchain-based implementations and vulnerabilities to adversarial manipulations are also underlined in this survey. The survey identifies a persistent trade-off between security and usability: higher accuracy is achieved through hybrid integrations such as elliptic curve cryptography, lightweight encryption, and blockchain; the challenges of scalability, efficiency, and real-world deployment are worth noting. Various attacks on biometric templates and systems have also been discussed, and privacy-preserving, efficient biometric matching in distributed environments is identified as a critical future direction.

## Figures and Tables

**Table 2:** Summary of the applications of fuzzy vaults across different datasets.

Reference	Year	Dataset	Technique Used	Performance
[53]	2004	Fingerprint dataset (100 images)	Fuzzy vault with adaptive alignment and imposter data points	Successful unlocking rate = 83%
[54]	2005	IBM-GTDB (100 images of minutiae)	Fuzzy vault with Galois fields, quantisation, CRC for error detection	GAR: 79%, FAR: 0%
[55]	2007	FVC2002-DB2, MSU-DBI	Fully automatic fuzzy vault with ridge endings, bifurcations, IPC algorithm	GAR: 91% (FVC2002-DB2), 82.5% (MSU-DBI)
[56]	2011	FVC fingerprint dataset, palm print dataset	Secure multimodal biometric authentication using fuzzy vault	No explicit quantitative metrics (qualitative evaluation)
[57]	2013	FVC2002-DB1, DB2	Delaunay triangle-based fuzzy vault with polar transformations	EER: 5.54% → 8.46% (DB1), 3.4% → 5.7% (DB2) after cancellable transformation; slight increase attributed to many-to-one mapping in polar transformation”
[58]	2020	FVC2002-DB1, DB2, DB3, DB4 (Set B)	Fuzzy vault with polynomial transformations and integral function transformation	GAR: 92% (Polynomial Degree 3), 90% (Degree 4), 85% (Degree 5)
[59]	2021	FVC2002-DB1, DB2, FVC2004-DB	PCA-based minutiae extraction for fuzzy vault	GAR = 91–94%, FAR = 0.72–0.98%, EER = 3.1–4.0% (p = 8); additional configs reported
[60]	2023	FVC2004-DB1, DB2, DB3, DB4	CNN-based biometric cryptography with fuzzy vault	Accuracy: 89.9%, PSNR: 9.8762 (DB4)
[61]	2007	BERC (990 iris images)	Fuzzy vault with ICA, K-means clustering, AES, Reed–Solomon codes, Lagrange’s interpolation	GAR: 99.225%, FRR: 0.775%, FAR: 0%
[13]	2008	BERC, CASIA V1.0, CASIA-IrisV3- Interval	Enhanced fuzzy vault with shift-matching, Iris Pseudo Codes	GAR: 99.675% (BERC), 89.78% (CASIA V1.0), 92.66% (CASIA-IrisV3-Interval)
[62]	2015	CASIA iris database	Fuzzy vault with 2D Gabor Wavelet Filter, Hamming and Euclidean distances	FAR: 0.01%, FRR: 0.15%
[63]	2016	CASIA v3-Interval, IITDv1	Multi-biometric fuzzy vault with Record-Specific Public Bijection	~97.0% GMR (at 0% FMR)
[64]	2019	CASIA v3-Interval	Alignment-free cancellable iris key binding using Bloom filter	GAR: 96.37%, FAR: 0%, hashed code length: 100
[65]	2019	e-passport, KDFE iris database, open-source fingerprint dataset	Multimodal biometrics with LSB steganography and fingerprint fuzzy vault	~0% FAR and FRR (SSIM ≈ 1.0, PSNR 53–56 dB for distributive mapping)
[66]	2021	CASIA v1, IITD v1	GLCM-based texture extraction with multi-key encryption	GAR: 99.86% (CASIA v1), 99.88% (IITD v1)
[67]	2020	EAR-IIT Delhi v1.0	Gabor filters with fuzzy vault, MMDA for dimension reduction	Accuracy: 98.83%
[68]	2023	AR face dataset	Dual-key fuzzy vault with PCA	Vault encryption/decryption time: 3.5 ms, recognition metrics not reported
[69]	2024	EEG dataset (28 participants), FVC2004 (80 fingerprint samples)	CNN-based feature extraction with score fusion	EER: 0.25% (EEG), 0.20% (fingerprint), 0.10% (multimodal)

**Table 3:** Summary of the comparative analysis of fuzzy vault parameters.

Aspect	Observations	Example Papers
Feature Representation	Minutiae-based methods are highly discriminative but alignment-sensitive; transformed (Delaunay, polar) and deep learning features improve robustness.	[55,57,60]
Polynomial Degree	Higher degree → stronger security but higher FRR (often exceeding 40% in high-degree configurations); lower degree → better usability	[57,58]
Chaff Points	Large chaff sets improve security but increase computational complexity and latency.	[54,55]
Robustness	Alignment-free and invariant representations improve tolerance to noise and distortion.	[53,61]
Computational Complexity	Early systems required up to 50 s for decoding; modern optimisations have reduced this to under a few seconds through feature filtering and efficient encoding.	[54,55]
Security vs. Usability	Strong cryptographic protection often leads to higher FRR and usability challenges.	[57,58]
Emerging Trends	Deep learning, multimodal fusion, and advanced cryptography improve performance but introduce computational and scalability challenges.	[56,60]

## Data Availability

No new data was generated.

## Abbreviations

The following abbreviations are used in this manuscript:

CNN	Convolutional Neural Network
CASIA	Chinese Academy of Sciences Institute of Automation
PCA	Principal Component Analysis
RS	Reed–Solomon
CRC	Cyclic Redundancy Check

## References

[1] Papathanasaki M., Maglaras L., Ayres N. (2022). Modern Authentication Methods: A Comprehensive Survey. AI Comput. Sci. Robot. Technol..

[2] Trader J. (2012). M2SYS Blog on Biometric Technology. http://blog.m2sys.com/biometric-hardware/iris-recognition-vs-retina-scanning-what-are-the-differences/.

[3] Patil S., Gudasalamani S., Iyer N.C. (2016). A survey on Iris recognition system. Proceedings of the 2016 International Conference on Electrical, Electronics, and Optimization Techniques.

[4] Daugman J.G. (1993). High confidence visual recognition of persons by a test of statistical independence. IEEE Trans. Pattern Anal. Mach. Intell..

[5] Belhumeur P.N. (2007). BIOMETRICS.

[6] Roy D.A., Urmila S.S. (2016). IRIS segmentation using Daughman’s method. Proceedings of the 2016 International Conference on Electrical, Electronics, and Optimization Techniques (ICEEOT).

[7] Hammouche R., Attia A., Akhrouf S., Akhtar Z. (2022). Gabor filter bank with deep autoencoder based face recognition system. Expert Syst. Appl..

[8] Suvorov N.V., Shleymovich M.P. (2020). Mathematical model of a biometric iris recognition system. Comput. Res. Model..

[9] Trokielewicz M., Czajka A., Maciejewicz P. (2020). Post-mortem iris recognition with deep-learning-based image segmentation. Image Vis. Comput..

[10] Sun Z., Tan T. (2009). Ordinal Measures for Iris Recognition. IEEE Trans. Pattern Anal. Mach. Intell..

[11] Monro D.M., Rakshit S., Zhang D. (2007). DCT-Based Iris Recognition. IEEE Trans. Pattern Anal. Mach. Intell..

[12] Abdullahi S.M., Sun S., Wang B., Wei N., Wang H. (2024). Biometric template attacks and recent protection mechanisms: A survey. Inf. Fusion.

[13] Lee Y.J., Park K.R., Lee S.J., Bae K., Kim J. (2008). A New Method for Generating an Invariant Iris Private Key Based on the Fuzzy Vault System. IEEE Trans. Syst. Man Cybern. Part B (Cybern.).

[14] Juels A., Sudan M. (2006). A Fuzzy Vault Scheme. Des. Codes Cryptogr..

[15] Yang W., Wang S., Hu J., Tao X., Li Y. (2024). Feature extraction and learning approaches for cancellable biometrics: A survey. CAAI Trans. Intell. Technol..

[16] Wieditz J., Pokern Y., Schuhmacher D., Huckemann S. (2022). Characteristic and necessary minutiae in fingerprints. J. R. Stat. Soc. Ser. C (Appl. Stat.).

[17] Neumann C., Champod C., Yoo M., Genessay T., Langenburg G. (2015). Quantifying the weight of fingerprint evidence through the spatial relationship, directions and types of minutiae observed on fingermarks. Forensic Sci. Int..

[18] Kulkarni J.V., Patil B.D., Holambe R.S. (2006). Orientation feature for fingerprint matching. Pattern Recognit..

[19] Ratha N.K.C., Connell J.H., Bolle R.M. (2001). Enhancing security and privacy in biometrics-based authentication systems. IBM Syst. J..

[20] Jin A.T.B., Ling D.N.C., Goh A. (2004). Biohashing: Two factor authentication featuring fingerprint data and tokenised random number. Pattern Recognit..

[21] Farooq F., Bolle R.M., Jea T.-Y., Ratha N. Anonymous and Revocable Fingerprint Recognition. Proceedings of the 2007 IEEE Conference on Computer Vision and Pattern Recognition.

[22] Bedari A., Wang S., Yang W. (2022). A Secure Online Fingerprint Authentication System for Industrial IoT Devices over 5G Networks. Sensors.

[23] Sun Y., Li H., Li N. (2023). A novel cancelable fingerprint scheme based on random security sampling mechanism and relocation bloom filter. Comput. Secur..

[24] Djebli H., Ait-Aoudia S., Michelucci D. (2023). Quantized random projections of SIFT features for cancelable fingerprints. Multimed. Tools Appl..

[25] Savvides M., Kumar B.V., Khosla P. Cancelable biometric filters for face recognition. Proceedings of the 17th International Conference on Pattern Recognition 2004, ICPR 2004.

[26] Oh B.-S., Toh K.-A., Choi K., Teoh A.B.J., Kim J. (2012). Extraction and fusion of partial face features for cancelable identity verification. Pattern Recognit..

[27] Faragallah O.S., Naeem E.A., El-Shafai W., Ramadan N., Ahmed H.E.-D.H., Elnaby M.M.A., Elashry I., El-Khamy S.E., El-Samie F.E.A. (2023). Efficient chaotic-Baker-map-based cancelable face recognition. J. Ambient. Intell. Humaniz. Comput..

[28] Xu Z., Shao Z., Shang Y., Li B., Ding H., Liu T. (2021). Fusing structure and color features for cancelable face recognition. Multimed. Tools Appl..

[29] Alhumyani H., El-Banby G.M., El-Sayed H.S., El-Samie F.E.A., Faragallah O.S. (2022). Efficient Generation of Cancelable Face Templates Based on Quantum Image Hilbert Permutation. Electronics.

[30] Ragendhu S.P., Thomas T. (2022). Cancelable biometric scheme based on dynamic salting of random patches. Multimed. Tools Appl..

[31] Hämmerle-Uhl J., Pschernig E., Uhl A. (2009). Cancelable Iris Biometrics Using Block Re-mapping and Image Warping. Information Security.

[32] Zuo J., Ratha N.K., Connell J.H. Cancelable iris biometric. Proceedings of the 2008 19th International Conference on Pattern Recognition.

[33] Rathgeb C., Breitinger F., Busch C. Alignment-free cancelable iris biometric templates based on adaptive bloom filters. Proceedings of the 2013 International Conference on Biometrics (ICB).

[34] Umer S., Dhara B.C., Chanda B. (2017). A novel cancelable iris recognition system based on feature learning techniques. Inf. Sci..

[35] Jang Y.K., Cho N.I. Deep Face Image Retrieval for Cancelable Biometric Authentication. Proceedings of the 2019 16th IEEE International Conference on Advanced Video and Signal Based Surveillance (AVSS).

[36] Kim B.-H., Pyun J.-Y. (2020). ECG Identification for Personal Authentication Using LSTM-Based Deep Recurrent Neural Networks. Sensors.

[37] Goodfellow I., Bengio Y., Courville A. (2016). Deep Learning.

[38] Abdellatef E., Ismail N.A., Abd Elrahman S.E.S.E., Ismail K.N., Rihan M., Abd El-Samie F.E. (2020). Cancelable multi-biometric recognition system based on deep learning. Vis. Comput..

[39] Sandhya M., Morampudi M.K., Pruthweraaj I., Garepally P.S. (2022). Multi-instance cancelable iris authentication system using triplet loss for deep learning models. Vis. Comput..

[40] Sakr A.S., Pławiak P., Tadeusiewicz R., Hammad M. (2022). Cancelable ECG biometric based on combination of deep transfer learning with DNA and amino acid approaches for human authentication. Inf. Sci..

[41] Kim J., Jung Y.G., Teoh A.B.J. (2022). Teoh Multimodal Biometric Template Protection Based on a Cancelable SoftmaxOut Fusion Network. Appl. Sci..

[42] Tarek M., Hamouda E., Abohamama A.S. (2022). Abohamama, Multi-instance cancellable biometrics schemes based on generative adversarial network. Appl. Intell..

[43] Sardar A., Umer S., Rout R.K., Khan M.K. (2023). A Secure and Efficient Biometric Template Protection Scheme for Palmprint Recognition System. IEEE Trans. Artif. Intell..

[44] Pintelas E., Livieris I.E., Barotsis N., Panayiotakis G., Pintelas P. (2021). An Autoencoder Convolutional Neural Network Framework for Sarcopenia Detection Based on Multi-frame Ultrasound Image Slices. Artificial Intelligence Applications and Innovations.

[45] Bamoriya P., Siddhad G., Khanna P., Ojha A. (2022). Cancelable Template Generation Using Convolutional Autoencoder and RandNet. Computer Vision and Image Processing.

[46] Dudhal A., Mathkar H., Jain A., Kadam O., Shirole M. (2019). Hybrid SIFT Feature Extraction Approach for Indian Sign Language Recognition System Based on CNN. Proceedings of the International Conference on ISMAC in Computational Vision and Bio-Engineering 2018 (ISMAC-CVB).

[47] Wulandari M., Chai R., Basari B., Gunawan D. (2024). Hybrid Feature Extractor Using Discrete Wavelet Transform and Histogram of Oriented Gradient on Convolutional-Neural-Network-Based Palm Vein Recognition. Sensors.

[48] Sawant G., Bharadi V., Prasad K., Jangid P. (2023). Hybrid Approach for Biometric Recognition: Integrating Custom Vector Quantization and CNN-Based Feature Extraction. Int. J. Intell. Syst. Appl. Eng..

[49] Wu S., Chen Y.C., Li X., Wu A.C., You J.J., Zheng W.S. An enhanced deep feature representation for person re-identification. Proceedings of the 2016 IEEE Winter Conference on Applications of Computer Vision (WACV).

[50] Kaur P., Kumar N. (2024). BRIEFBCS: Binary robust independent elementary features based fuzzy vault scheme in BCS. Clust. Comput..

[51] Nguyen M.T., Truong Q.H., Dang T.K. (2016). Enhance fuzzy vault security using nonrandom chaff point generator. Inf. Process. Lett..

[52] Nagar A., Nandakumar K., Jain A.K. Securing fingerprint template: Fuzzy vault with minutiae descriptors. Proceedings of the 2008 19th International Conference on Pattern Recognition.

[53] Yang S., Verbauwhede I. Secure fuzzy vault-based fingerprint verification system. Proceedings of the Conference Record of the Thirty-Eighth Asilomar Conference on Signals, Systems and Computers 2004.

[54] Uludag U., Pankanti S., Jain A.K. (2005). Fuzzy Vault for Fingerprints. Audio- and Video-Based Biometric Person Authentication.

[55] Nandakumar K., Jain A.K., Pankanti S. (2007). Fingerprint-Based Fuzzy Vault: Implementation and Performance. IEEE Trans. Inf. Forensics Secur..

[56] Brindha V.E. Biometric template security using fuzzy vault. Proceedings of the 2011 IEEE 15th International Symposium on Consumer Electronics (ISCE).

[57] Yang W., Hu J., Wang S. A Delaunay triangle group based fuzzy vault with cancellability. Proceedings of the 2013 6th International Congress on Image and Signal Processing (CISP).

[58] Mehmood R., Selwal A. (2020). Polynomial Based Fuzzy Vault Technique for Template Security in Fingerprint Biometrics. Int. Arab. J. Inf. Technol..

[59] Baghel V.S., Prakash S., Agrawal I. (2021). An enhanced fuzzy vault to secure the fingerprint templates. Multimed. Tools Appl..

[60] Kaur P., Kumar N. Biometric Cryptosystem with Deep Learning: A New Frontier in Security. Proceedings of the 2023 International Conference on Advances in Power, Signal, and Information Technology (APSIT).

[61] Lee Y.J., Bae K., Lee S.J., Park K.R., Kim J. (2007). Biometric Key Binding: Fuzzy Vault Based on Iris Images. Advances in Biometrics.

[62] Arora A., Divekar R. (2015). Bio-Cryptosystem Using Fuzzy Vault Scheme. Int. J. Recent Innov. Trends Comput. Commun..

[63] Rathgeb C., Tams B., Wagner J., Busch C. (2016). Unlinkable improved multi-biometric iris fuzzy vault. EURASIP J. Inf. Secur..

[64] Chai T.-Y., Goi B.-M., Tay Y.-H., Jin Z. (2019). A New Design for Alignment-Free Chaffed Cancelable Iris Key Binding Scheme. Symmetry.

[65] Bobkowska K., Nagaty K., Przyborski M. (2019). Incorporating iris, fingerprint and face biometrics for fraud prevention in e-passports using fuzzy vault. IET Image Process..

[66] Taha M.A., Ahmed H.M. (2021). A fuzzy vault development based on iris images. EUREKA Phys. Eng..

[67] Kavipriya A., Arunachalam M. (2020). New Intuition on Ear Authentication with Gabor Filter Using Fuzzy Vault. 3C Tecnol. Glosas Innov. Apl. A La Pyme.

[68] Choi J., Lee J., Kim A. (2023). An Efficient Confidence Interval-Based Dual-Key Fuzzy Vault Scheme for Operator Authentication of Autonomous Unmanned Aerial Vehicles. Appl. Sci..

[69] Nadamau M.S., Musa K.I., Galoji S.I. (2024). Multimodal Biometric Identification System Based on EEG (Electroencephalograph) and Fingerprint with Template Protection. Anchor Univ. J. Sci. Technol..

[70] Majbour S., Barbier M., Le Bars J.-M. Fuzzy Vault Security Enhancement Avoid Statistical Biases. Proceedings of the 21st International Conference on Security and Cryptography SECRYPT.

[71] Sharma S., Saini A., Chaudhury S. (2024). Multimodal biometric user authentication using improved decentralized fuzzy vault scheme based on blockchain network. J. Inf. Secur. Appl..

[72] Maurya P.K., Sandhya M., Prasad I.H., SuryanarayanaRaju N.V. (2025). Enhancing fingerprint template security using elliptic curve cryptography and fuzzy vault encoding. J. Inf. Secur. Appl..

[73] Sulavko A., Panfilova I., Inivatov D., Lozhnikov P., Vulfin A., Samotuga A. (2025). Biometric-based key generation and user authentication using voice password images and neural fuzzy extractor. Appl. Syst. Innov..

[74] Mohan S., Kumari V.S., Pillai N.M., Aswathy R.H., Santhoshkumar S.P., Arora P. (2025). A Multi-Layered Cryptographic Model for Biometric Data Security Using Fuzzy Vault and Lightweight AES Variants. Proceedings of the 2025 5th International Conference on Emerging Research in Electronics, Computer Science and Technology (ICERECT).

[75] Bauspieß P., Silde T., Poljuha M., Tullot A., Costache A., Rathgeb C., Kolberg J., Busch C. (2024). Brake: Biometric resilient authenticated key exchange. IEEE Access.

[76] Zhang B., Hu S., Wang Z., Ren K. (2025). Fuzzy Vault Revisited–Enabling Privacy-preserving Multi-modal Biometric Authentication. IEEE Trans. Dependable Secur. Comput..

[77] Dellys H.N., Sliman L., Morris B.T., Benatchba K. (2024). On Efficiency of Square-Boundaries Chaff Points Generation with Composite Representation in Fingerprint Fuzzy Vault. IEEE Access.

[78] Geng S., Giannopoulou G., Kabir-Querrec M. Privacy protection in distributed fingerprint-based authentication. Proceedings of the 18th ACM Workshop on Privacy in the Electronic Society.

[79] Zhang M., Marin E., Oswald D., Singelée D. (2021). FuzzyKey: Comparing fuzzy cryptographic primitives on resource-constrained devices. International Conference on Smart Card Research and Advanced Applications.

[80] Merkle J., Niesing M., Schwaiger M., Ihmor H., Korte U. (2010). Performance of the fuzzy vault for multiple fingerprints. arXiv.

[81] Geißner H., Rathgeb C. (2025). Closing the performance gap in biometric cryptosystems: A deeper analysis on unlinkable fuzzy vaults. arXiv.

[82] Lafkih M., Mikram M., Ghouzali S., El Haziti M., Aboutajdine D. (2012). Biometric cryptosystems based fuzzy vault approach: Security analysis. Proceedings of the Second International Conference on the Innovative Computing Technology (INTECH 2012).

[83] Abiega-L’Eglisse A.F.D., Otero M.R., Hernández V.A., Gallegos-Garcia G., Nakano-Miyatake M. (2022). A new fuzzy vault based biometric system robust to brute-force attack. Comput. Y Sist..

[84] Kholmatov A., Yanikoglu B. (2008). Realization of correlation attack against the fuzzy vault scheme. Proceedings of the Security, Forensics, Steganography, and Watermarking of Multimedia Contents X.

[85] Du X., Zhu J., Zhou J.-Z., Pun C.-M., Lin Z., Wu C., Chen Z., Luo J. (2025). Dp-trae: A dual-phase merging transferable reversible adversarial example for image privacy protection. IEEE Trans. Dependable Secur. Comput..

[86] Zhou Y., Luo T., He Z., Jiang G., Xu H., Chang C.-C. (2024). CAISFormer: Channel-wise attention transformer for image steganography. Neurocomputing.

[87] Rathgeb C., Merkle J., Scholz J., Tams B., Nesterowicz V. (2022). Deep face fuzzy vault: Implementation and performance. Comput. Secur..

[88] Rathgeb C., Tams B., Merkle J., Nesterowicz V., Korte U., Neu M. Multi-Biometric Fuzzy Vault based on Face and Fingerprints. Proceedings of the 2023 IEEE International Joint Conference on Biometrics (IJCB).

[89] Ali A., Baghel V.S., Prakash S. (2022). An Alignment-Free Fingerprint Template Protection Technique Based on Minutiae Triplets. Recent Trends in Image Processing and Pattern Recognition.

[90] Pradel G., Mitchell C. Privacy-Preserving Biometric Matching Using Homomorphic Encryption. Proceedings of the 2021 IEEE 20th International Conference on Trust, Security and Privacy in Computing and Communications (TrustCom).

[91] Xu G., Fan X., Xu S., Cao Y., Zhang K., Kang J., Niyato D. (2025). Towards authenticated encrypted search with constant trapdoor for mobile cloud systems. IEEE Trans. Mob. Comput..

[92] Xu W., Deng J., Yu J., Mao S., Li Y., Peng Z., Xiao B. (2025). Blockchain-based verifiable decentralized identity for intelligent flexible manufacturing. IEEE Internet Things J..

